# Supplementary material for: Tactile modulation of memory and anxiety requires dentate granule cells along the dorsoventral axis
Source: Nat Commun. 2020 Nov 27;11:6045. doi: 10.1038/s41467-020-19874-8 (PMC7695841; doi:10.1038/s41467-020-19874-8)
Supplement: Supplementary file 1 — Supplementary Information [file 41467_2020_19874_MOESM1_ESM.pdf]

Supplementary Information for:

Tactile Modulation of Memory and Anxiety Requires Dentate Granule Cells along the Dorsoventral Axis

Chi Wang, Hui Liu, Kun Li, Zhen-Zhen Wu, Chen Wu, Jing-Ying Yu, Qian Gong, Ping Fang, Xing-Xing Wang, Shu-Min Duan, Hao Wang, Yan Gu, Ji Hu, Bing-Xing Pan, Mathias V. Schmidt, Yi-Jun Liu, and Xiao-Dong Wang

Correspondence: Dr. Xiao-Dong Wang, xiaodongwang@zju.edu.cn

Including:

1. Supplementary Figures 1-13

2. Supplementary Tables 1-2

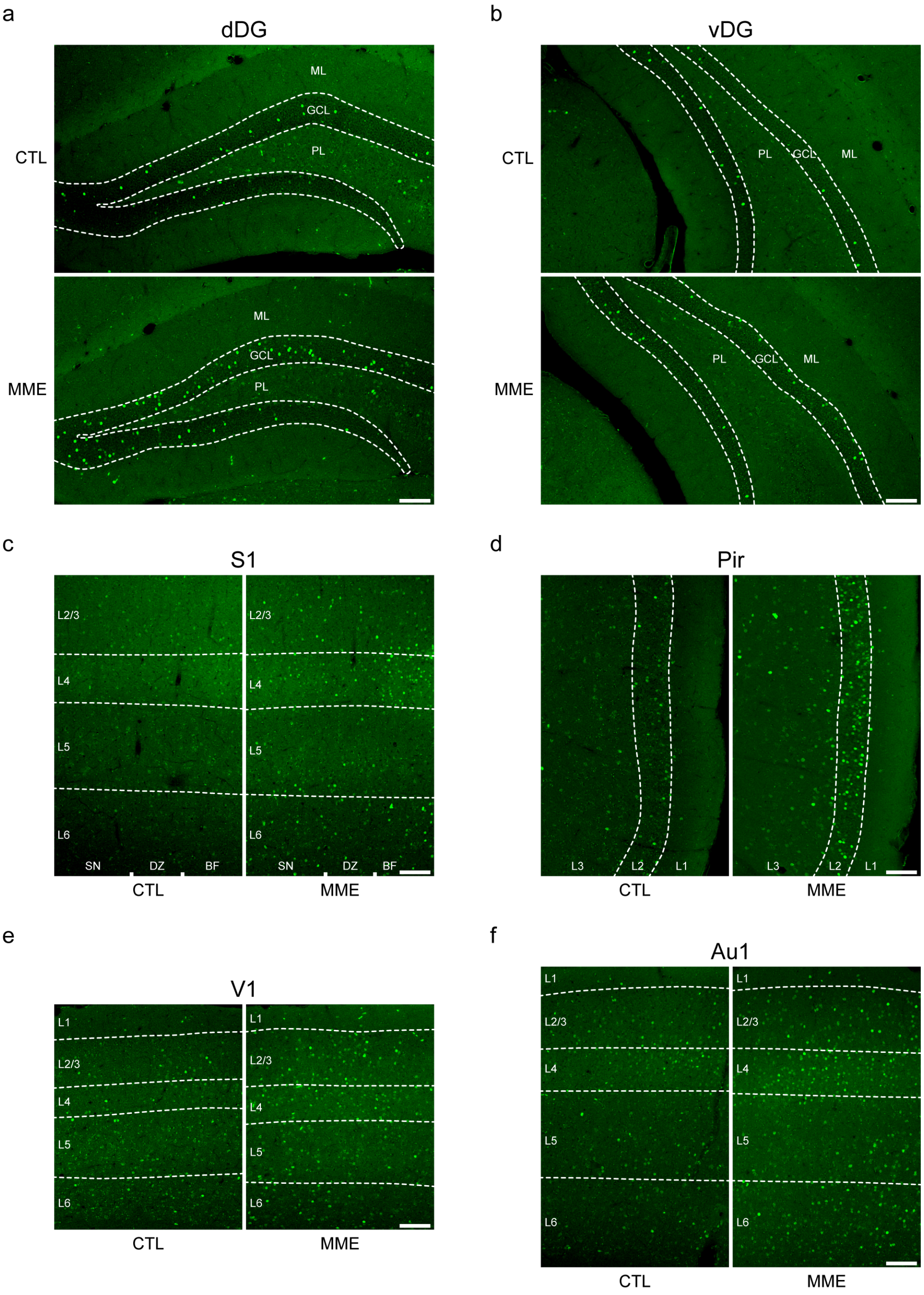

Supplementary Fig. 1 Neuronal activation in the dentate gyrus and primary sensory cortices after multimodal enrichment. **a-f** Representative images show c-fos immunostaining in the dorsal dentate gyrus (dDG, **a**), ventral dentate gyrus (vDG, **b**), primary somatosensory cortex (S1, **c**), piriform cortex (Pir, **d**), primary visual cortex (V1, **e**), and primary auditory cortex (Au1, **f**) of the control (CTL) and multimodal enrichment (MME) groups. Associated data are

presented in **Fig. 1c**-**e**. Detailed statistics are provided in Supplementary Table 2. Source data are provided as a Source Data file. ML, molecular layer; GCL, granule cell layer; PL, polymorphic layer; L, layer; SN, shoulder/neck region; DZ, dysgranular zone; BF, barrel field. Scale bars = 100 μm.

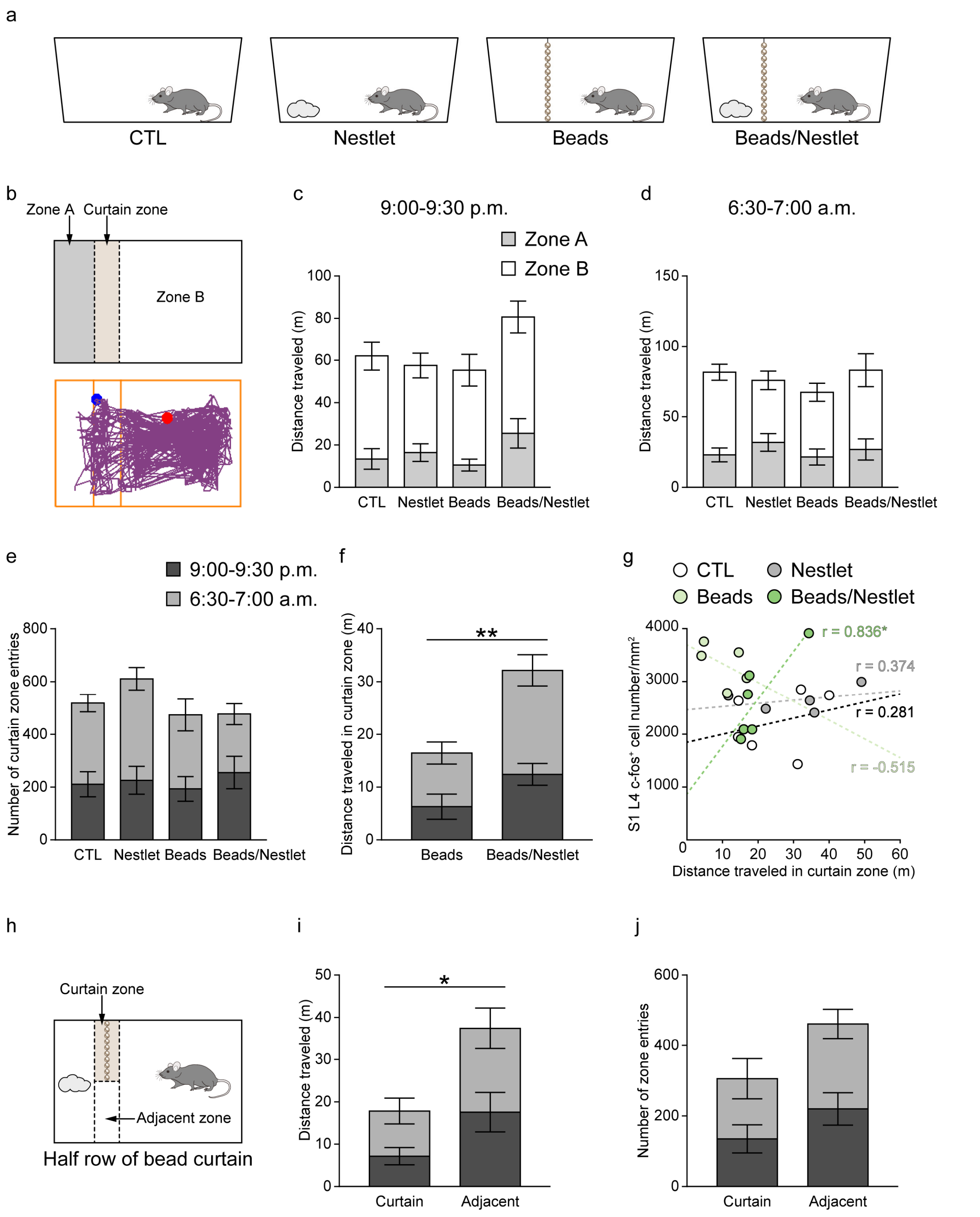

Supplementary Fig. 2 Establishment of a mouse model of unimodal tactile experience enrichment. **a** Illustrations of the setup of cages. Mice were singly housed in standard cages (CTL) or cages provided with cotton nesting material (Nestlet), a row of glass bead curtain (Beads), or both (Beads/Nestlet). **b** The upper panel shows a schematic cage top view. Each

cage was divided into three zones: zone A (where the nesting material was provided in the Nestlet and Beads/Nestlet cages), curtain zone (where the bead curtain was placed in the Beads and Beads/Nestlet cages), and zone B (where food and water were provided). The lower panel shows a representative 30-min track plot of a mouse exploring a Beads/Nestlet cage. **c**, **d** The experiment was started at 1 h before the dark phase on day 1. Home cage activity was monitored at 9:00-9:30 p.m. on day 2 (**c**) and 6:30-7:00 a.m. on day 3 (**d**). In the dark phase on days 2 and 3, all groups of mice explored zone A or B similarly. **e** The number of visits to the curtain zone did not differ among groups. **f** Compared to mice housed with the bead curtain only, mice with both a bead curtain and nesting material traveled more distance in the curtain zone. **g** A significant correlation between the density of c-fos$^+$ cells in S1 layer 4 and exploration in the curtain zone was observed only in the Beads/Nestlet group. Therefore, the Beads/Nestlet setup was used in all subsequent experiments. **h** A half row of bead curtain consisting of 10 bead strings was provided on one side of the cage to examine whether mice showed avoidance of the bead curtain. Exploration of the curtain zone and the adjacent equal-sized zone was analyzed. **i**, **j** Although mice traveled less distance in the curtain zone than the adjacent zone (**i**), the number of visits to two zones was comparable (**j**), indicating that the bead curtain is not an aversive stimulus for mice. n = 6-7 mice per group for (**c**-**e**), 6 mice per group for (**f** and **g**) and 6 mice for (**h**-**j**). $^*P < 0.05$, $^{**}P < 0.01$. Data in bar charts are presented as mean ± SEM. Detailed statistics are provided in Supplementary Table 2. Source data are provided as a Source Data file.

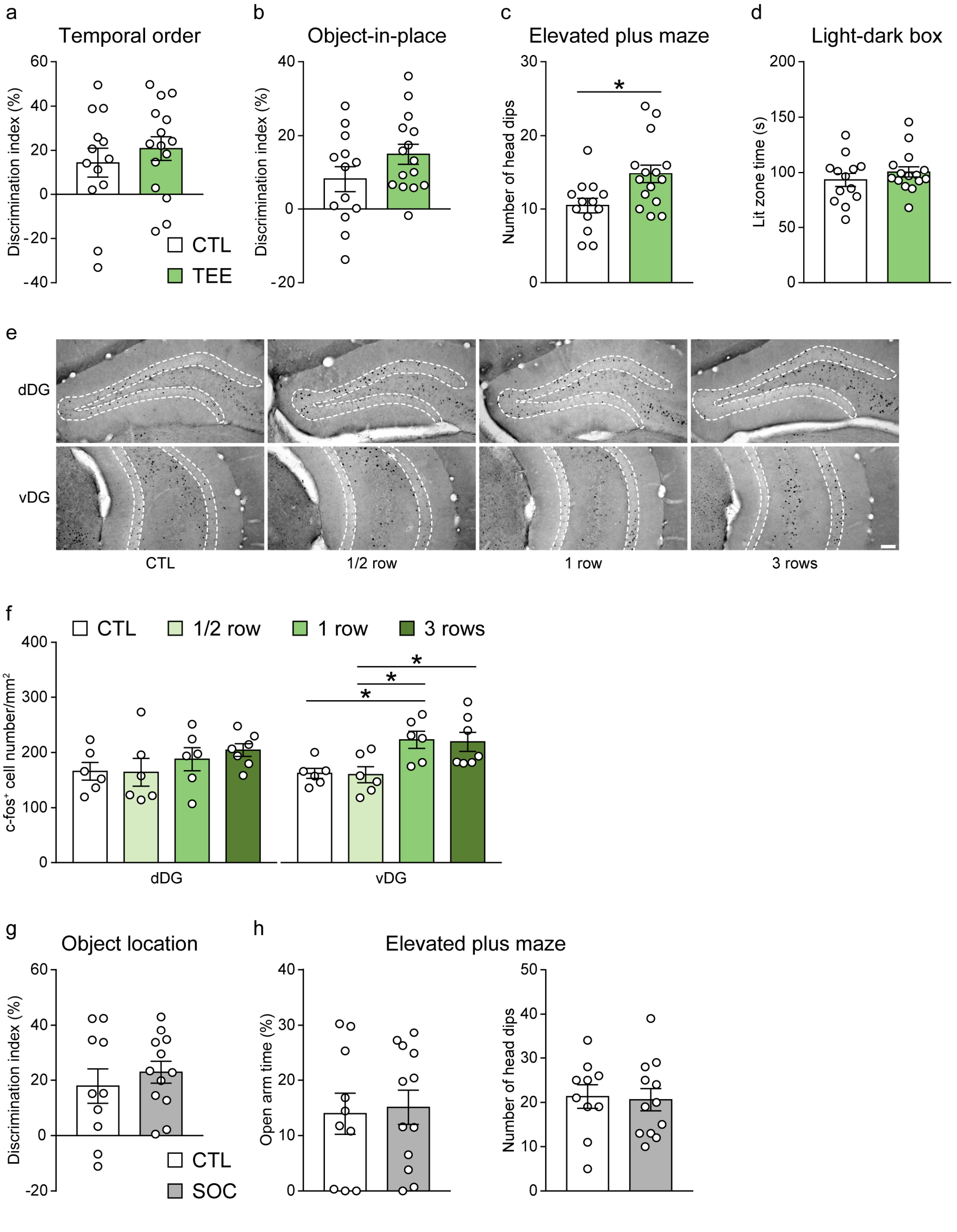

Supplementary Fig. 3 Effects of tactile enrichment, varied rows of bead curtain and social housing on memory, anxiety-related behavior or DG neuron activation. **a** Control (CTL) mice and mice with tactile experience enrichment (TEE) showed comparable temporal order memory performance. **b** Tactile enrichment did not alter performance in the object-in-place task. **c** TEE mice exhibited reduced anxiety-related behavior, as shown by more head dips in

the elevated plus maze than CTL mice. **d** CTL and TEE mice spent similar amount of time in the brightly illuminated chamber of the light-dark box. **e** Representative photomicrographs show c-fos immunostaining in dDG and vDG from mice housed under standard conditions or with a half row, 1 row or 3 rows of bead curtain. Dashed regions indicate dentate granule cell layer. Scale bar = 100 μm. **f** No difference in the density of activated dDG neurons was observed among groups, whereas mice housed with 1 row or 3 rows of bead curtain had increased neuronal activation in vDG. **g, h** Compared to singly housed controls, socially housed mice (SOC) showed similar performance in the object location task (**g**) and the elevated plus maze test (**h**). n = 13-15 mice per group for (**a-d**), 6-7 mice per group for (**f**) and 10-12 mice per group for (**g** and **h**). $*P < 0.05$. Data are presented as mean ± SEM. Detailed statistics are provided in Supplementary Table 2. Source data are provided as a Source Data file.

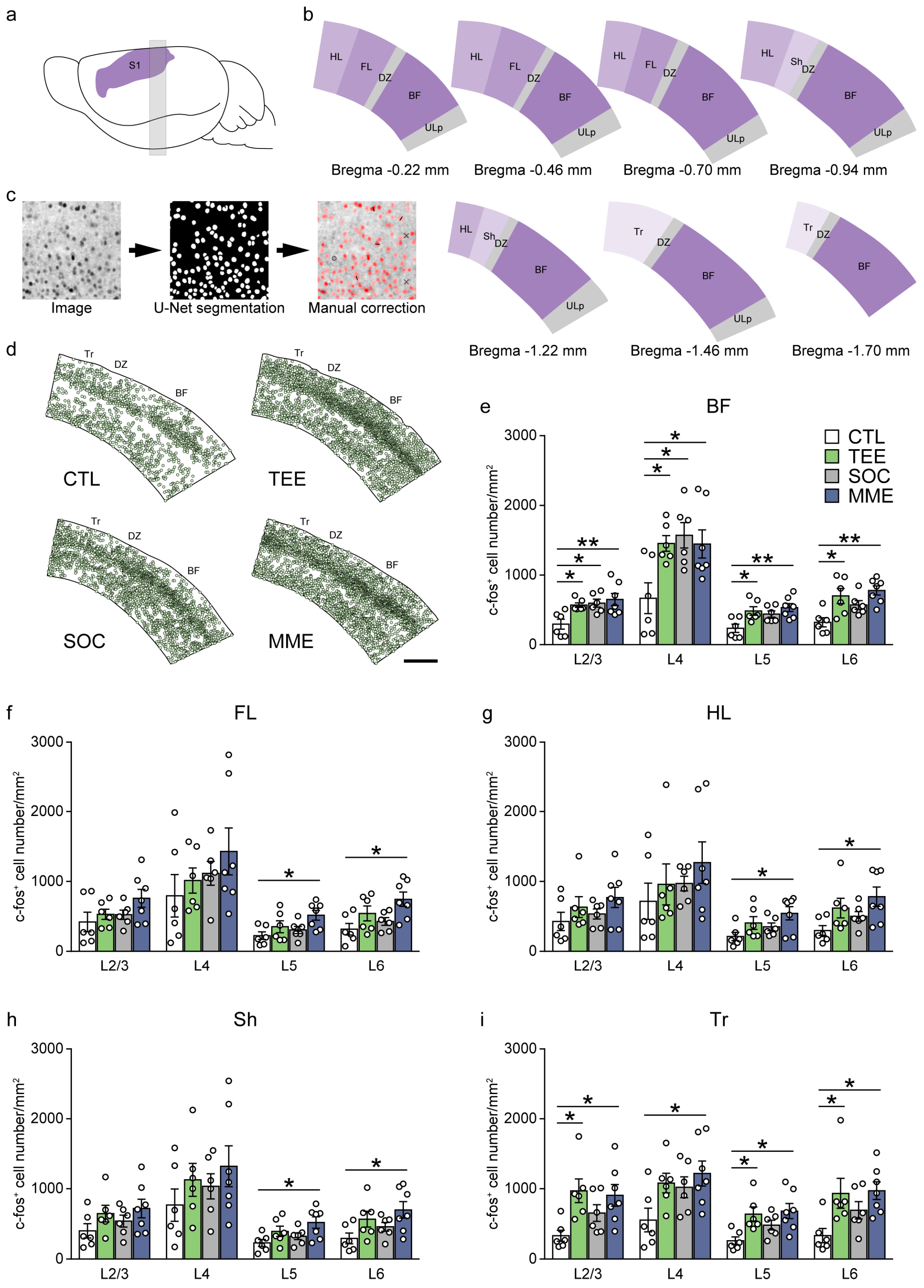

Supplementary Fig. 4 Effects of tactile enrichment, social housing and multimodal enrichment on neuronal activation in S1 subregions. **a** Serial coronal sections from the shaded region were selected for S1 c-fos^{+} neuron quantification. **b** Diagrams showing S1 subregions

at different coronal planes within the shaded area in (**a**). Subregions with relatively distinguishable borders (marked with purple colors) were included for analysis. HL, hindlimb; FL, forelimb; DZ, dysgranular zone; BF, barrel field; ULp, upper lip; Sh, shoulder; Tr, trunk. **c** The pipeline for image segmentation and manual correction of segmented c-fos$^+$ neurons. The image was imported to ImageJ and segmented by the U-Net Segmentation plugin, followed by manual labeling of unrecognized neurons (circle), removal of misrecognized neurons (crosses), and splitting of merged neurons (lines). **d** Representative plots of c-fos$^+$ neurons in the S1 (~1.7 mm posterior to bregma) of CTL (n = 6), TEE (n = 6), SOC (n = 6), and MME (n = 7) mice. Scale bar = 500 µm. **e** In the S1 barrel field, 10 days of TEE or MME increased neuronal activation in layers 2-6, while SOC increased neuronal activation in layers 2-4. **f-h** In the forelimb (**f**), hindlimb (**g**) and shoulder (**h**) regions of S1, MME increased c-fos$^+$ neuron density in layers 5 and 6. **i** In the trunk region of S1, TEE and MME increased c-fos$^+$ neuron density in specific layers. *$P < 0.05$, **$P < 0.01$. Data are presented as mean ± SEM. Detailed statistics are provided in Supplementary Table 2. Source data are provided as a Source Data file.

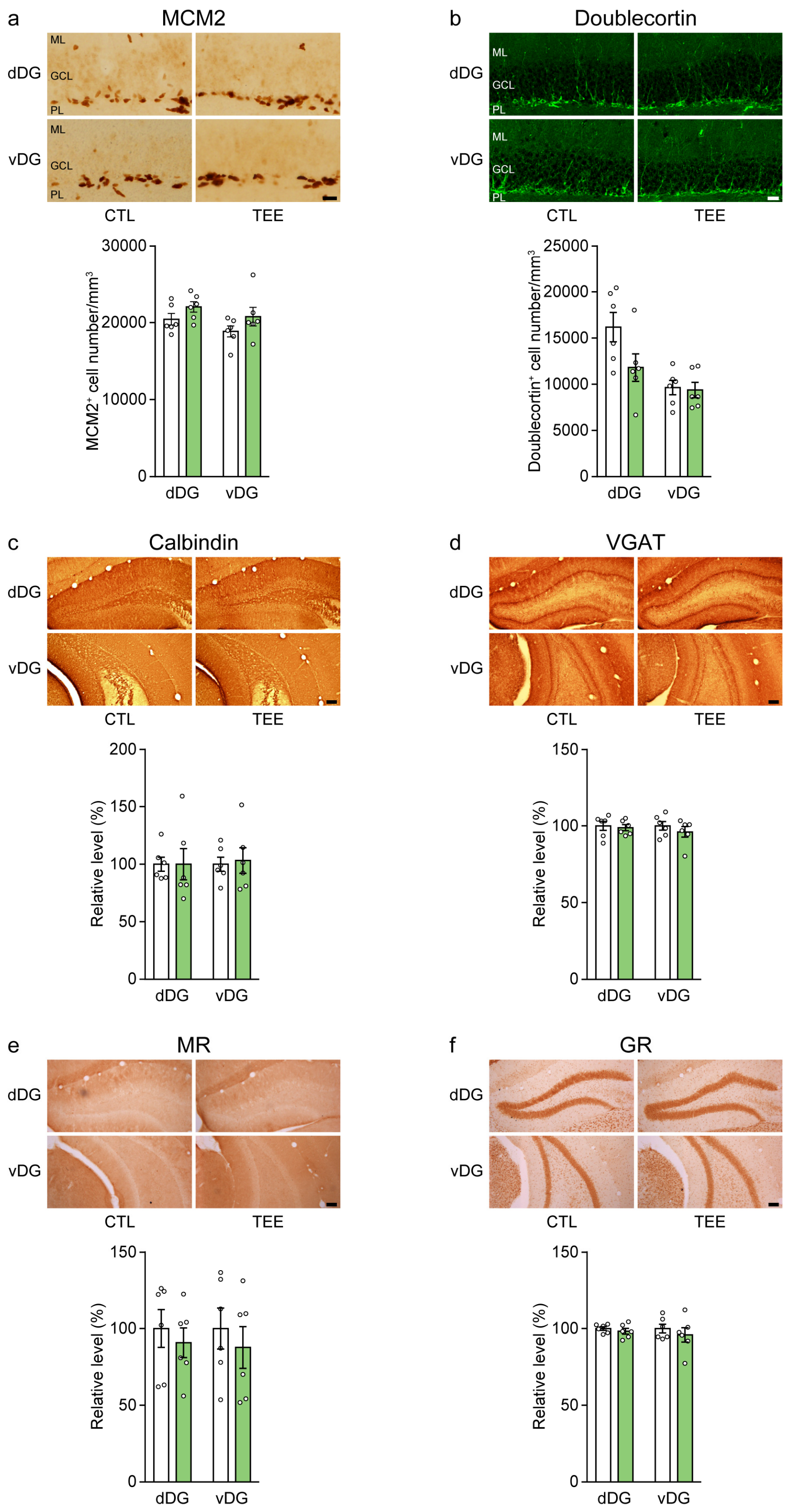

Supplementary Fig. 5 Effects of repeated tactile enrichment on the expression of molecules related to neurogenesis, inhibitory synapse and stress response in dDG and vDG. **a** Tactile

enrichment for 20 days did not change the density of minichromosome maintenance complex component 2 (MCM2)-expressing proliferating cells in the DG. **b** The density of doublecortin^{+} differentiating cells was comparable between groups. **c** The immunoreactivity of calbindin, a marker of mature dentate granule cells, remained unchanged in dDG and vDG after TEE. **d**-**f** Protein levels of vesicular GABA transporter (VGAT, **d**), mineralocorticoid receptor (MR, **e**), and glucocorticoid receptor (GR, **f**) were comparable between groups. Scale bars: 20 μm for (**a** and **b**) and 100 μm for (**c**-**f**). n = 6 mice per group. Data are presented as mean ± SEM. Detailed statistics are provided in Supplementary Table 2. Source data are provided as a Source Data file.

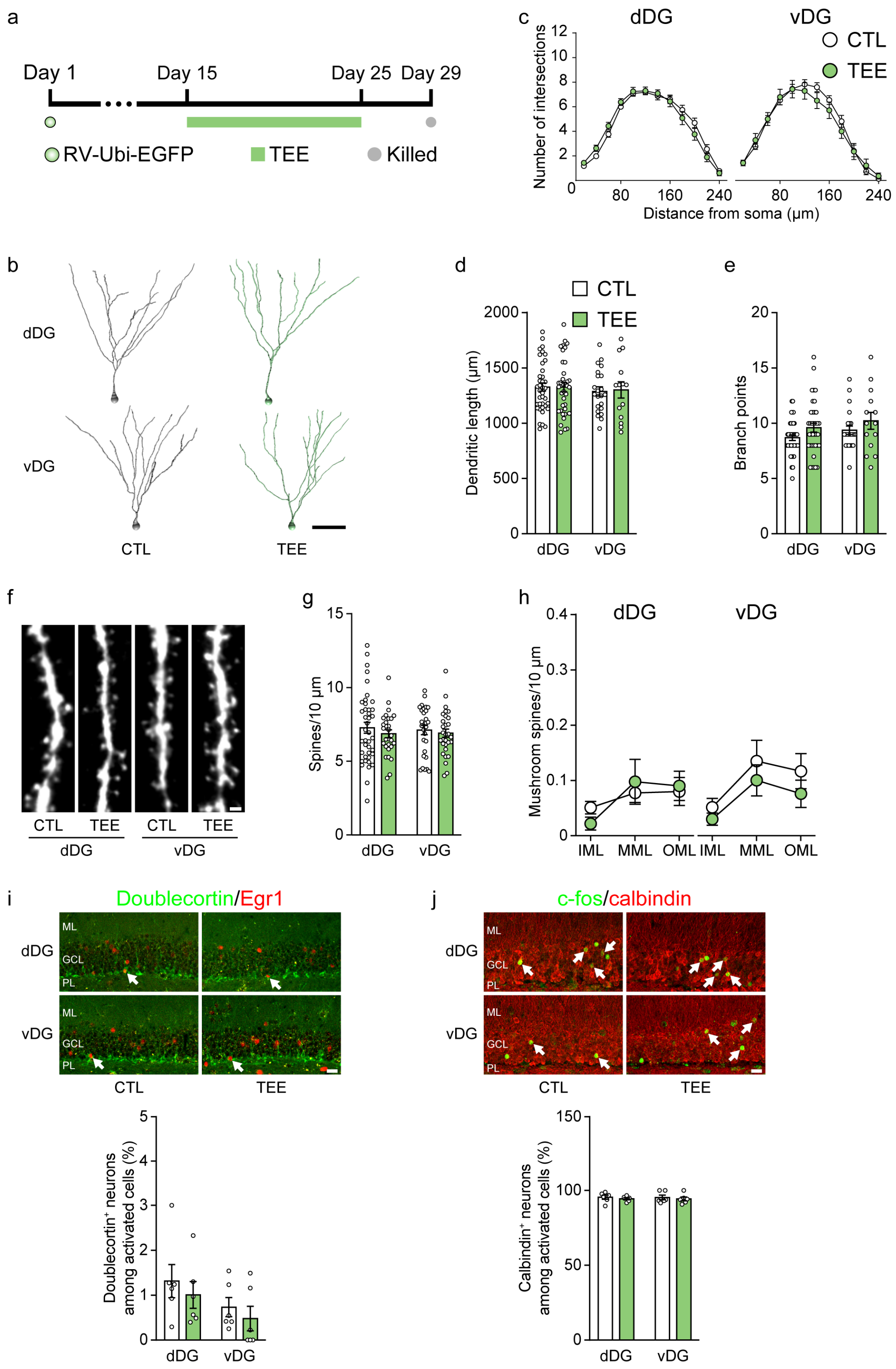

Supplementary Fig. 6 Effects of tactile enrichment on the morphological maturation of adult-born granule cells and the activation of neurons in dDG and vDG. **a** The timeline of experiment. EGFP-expressing retrovirus (RV) was used to label adult-born dentate granule

cells. **b** Representative reconstructions of 4-week-old dDG and vDG granule cells from the CTL and TEE groups. **c-e** The number of dendritic intersections at concentric circles (**c**), total dendritic length (**d**) and branch point number (**e**) were comparable between CTL and TEE groups. **f** Representative images of dendritic segments in the outer molecular layer of dDG and vDG. **g, h** Total spine density (**g**) and mushroom spine density in the inner, medial and outer molecular layers (abbreviated as IML, MML and OML) of the DG (**h**) were similar between groups. **i** The percentage of doublecortin^{+} neurons among all activated dentate granule cells immunostained with early growth response 1 (Egr1) was comparable between groups. Arrows indicate doublecortin and Egr1 double-labeled cells. **j** The percentage of calbindin^{+} neurons among all c-fos^{+} granule cells was comparable between groups. Arrows indicate calbindin and c-fos double-labeled cells. Scale bars: 50 μm for (**b**), 1 μm for (**f**) and 20 μm for (**i** and **j**). n = 6-8 mice per group for (**c-h**) and 6 mice per group for (**i** and **j**). Data are presented as mean ± SEM. Detailed statistics are provided in Supplementary Table 2. Source data are provided as a Source Data file.

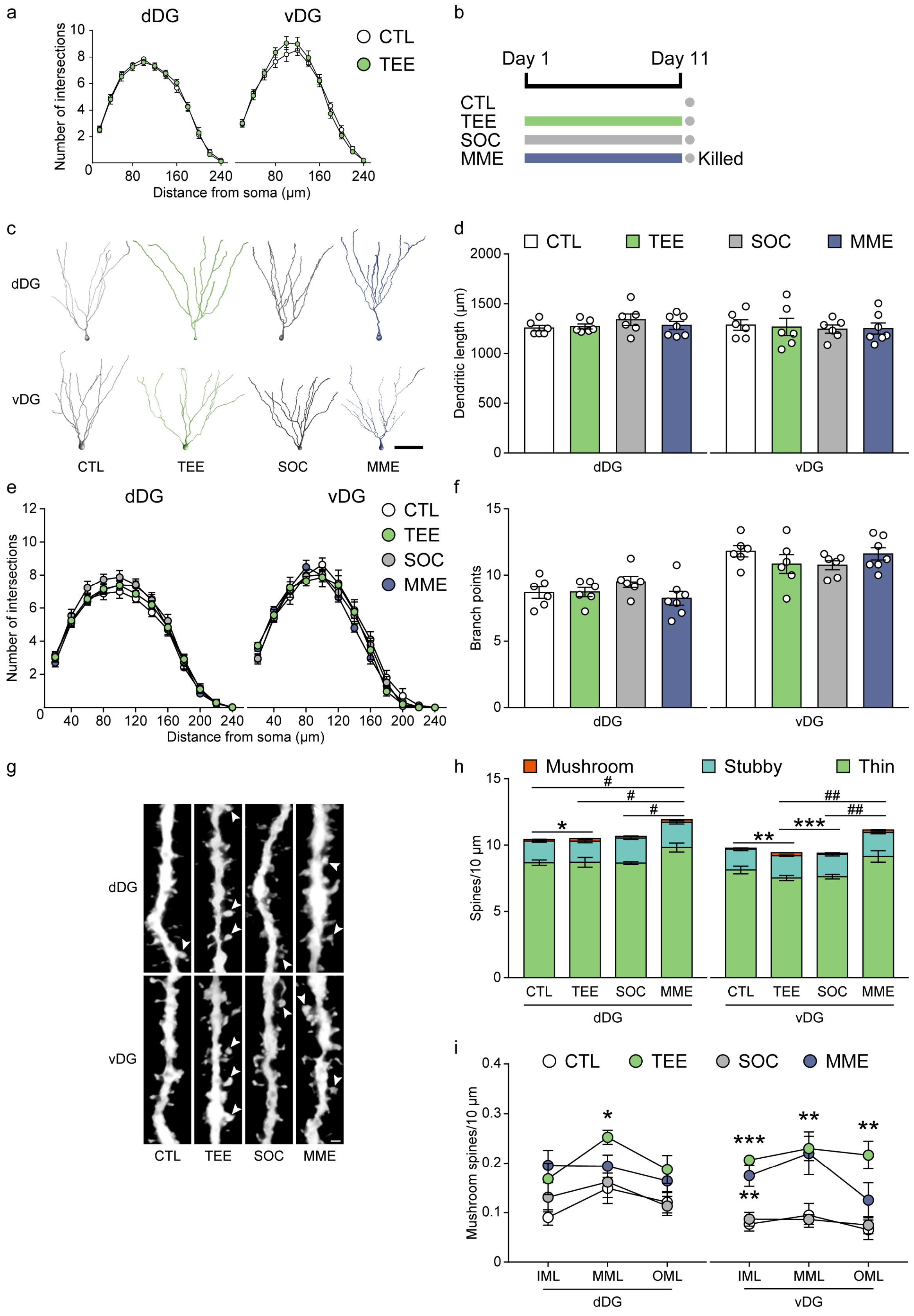

Supplementary Fig. 7 Effects of tactile enrichment, social housing and multimodal

enrichment on DG neuron structural plasticity as revealed by Golgi-Cox staining. **a** The number of dendritic intersections at concentric circles was comparable between CTL and TEE groups (corresponds to **Fig. 4b-d**). **b** The timeline of experiment (corresponds to **c-i**). **c** Representative reconstructions of dDG and vDG granule cells from the CTL, TEE, SOC, and MME groups. Scale bar = 50 μm. **d-f** Total dendritic length (**d**), dendritic intersections (**e**) and branch point number (**f**) were comparable among groups. **g** Representative images of dendritic segments in the outer molecular layer of dDG and vDG. Arrowheads indicate mushroom spines. Scale bar = 1 μm. **h** In the dDG, mushroom spines density was increased in the TEE group compared to the CTL group, while thin spine density in the MME group was higher compared to other groups. In the vDG, mushroom spine density was increased in the TEE group compared to CTL and SOC groups, while thin spine density in the MME group was higher compared to TEE and SOC groups. **i** Tactile enrichment and multimodal enrichment increased mushroom spine density in specific sublayers of dDG and vDG. n = 8 mice per group for (**a**) and 6-7 mice per group for (**d-i**). $^{*}P < 0.05$, $^{**}P < 0.01$, $^{***}P < 0.001$ for pairwise comparisons of mushroom spine density between groups. $^{\#}P < 0.05$, $^{\#\#}P < 0.01$ for pairwise comparisons of thin spine density between groups. Data are presented as mean ± SEM. Detailed statistics are provided in Supplementary Table 2. Source data are provided as a Source Data file.

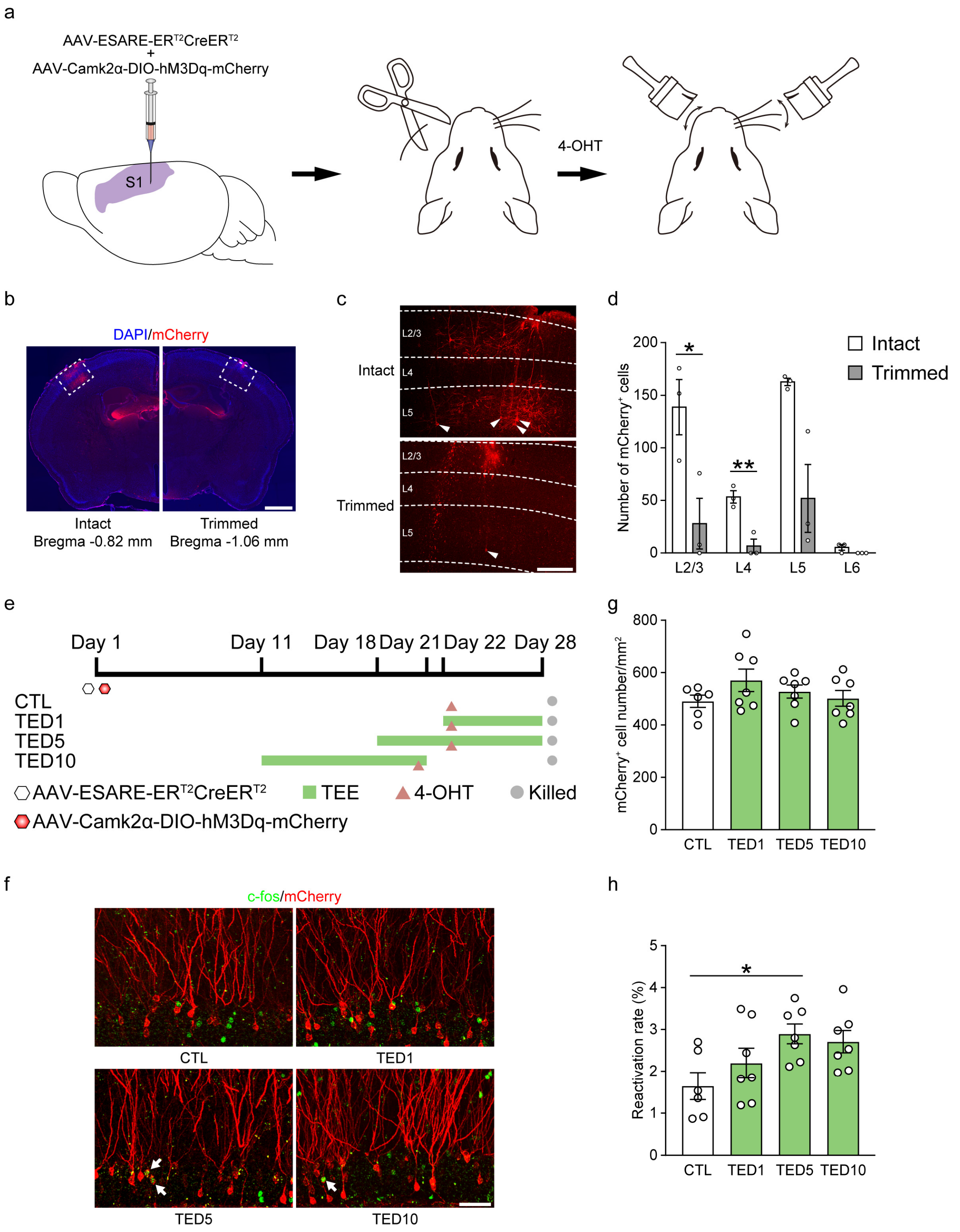

Supplementary Fig. 8 The validation of viral vectors and reactivation analysis of conditionally labeled dDG neurons. **a** Illustration of the strategy to test the specificity of AAV-ESARE-ER^{T2}CreER^{T2}. Viral vectors were bilaterally injected to the adult mouse S1. After 4 weeks of recovery, vibrissae on one side of the face were trimmed, followed by a

subcutaneous injection of 4-hydroxytamoxifen (4-OHT). At 30 min after 4-OHT injection, whisker pads on both sides were brushed for 15 min. **b** Representative images show mCherry expression in the contralateral (intact) and ipsilateral (trimmed) S1 to the side without whisker trimming. Boxed regions were imaged at a higher magnification and are presented in **(c)**. **c** Representative images show tactile stimulation-activated neurons in S1 layers. Arrows indicate mCherry$^+$ pyramidal-shaped neurons in S1 layer 5. **d** Stimulation of intact whiskers significantly increased the number of mCherry-labeled neurons in the contralateral S1, whereas neurons in the contralateral S1 to the trimmed side were rarely labeled. **e** The timeline of the dDG neuron reactivation experiment. Following viral delivery to the dDG, neurons that were active under home-cage conditions (CTL) or on day 1 (TED1), day 5 (TED5) or day 10 (TED10) of tactile enrichment were labeled by 4-OHT. Note that in the TED1 group, 4-OHT was inject at 12 h after enrichment started. Mice were killed at 6 or 8 days after the 4-OHT injection. **f** Representative images show conditionally labeled dDG neurons (mCherry$^+$) and active neurons 6 or 8 days later (c-fos$^+$). **g** The density of mCherry-labeled dDG neurons was comparable among groups. **h** The reactivation rate of TED5 neurons was higher compared to CTL neurons. Scale bars: 1 mm for (**b**), 200 μm for (**c**) and 50 μm for (**f**). n = 3 mice for (**d**) and 6-7 mice per group for (**g** and **h**). $*P < 0.05$, $**P < 0.01$. Data are presented as mean ± SEM. Detailed statistics are provided in Supplementary Table 2. Source data are provided as a Source Data file.

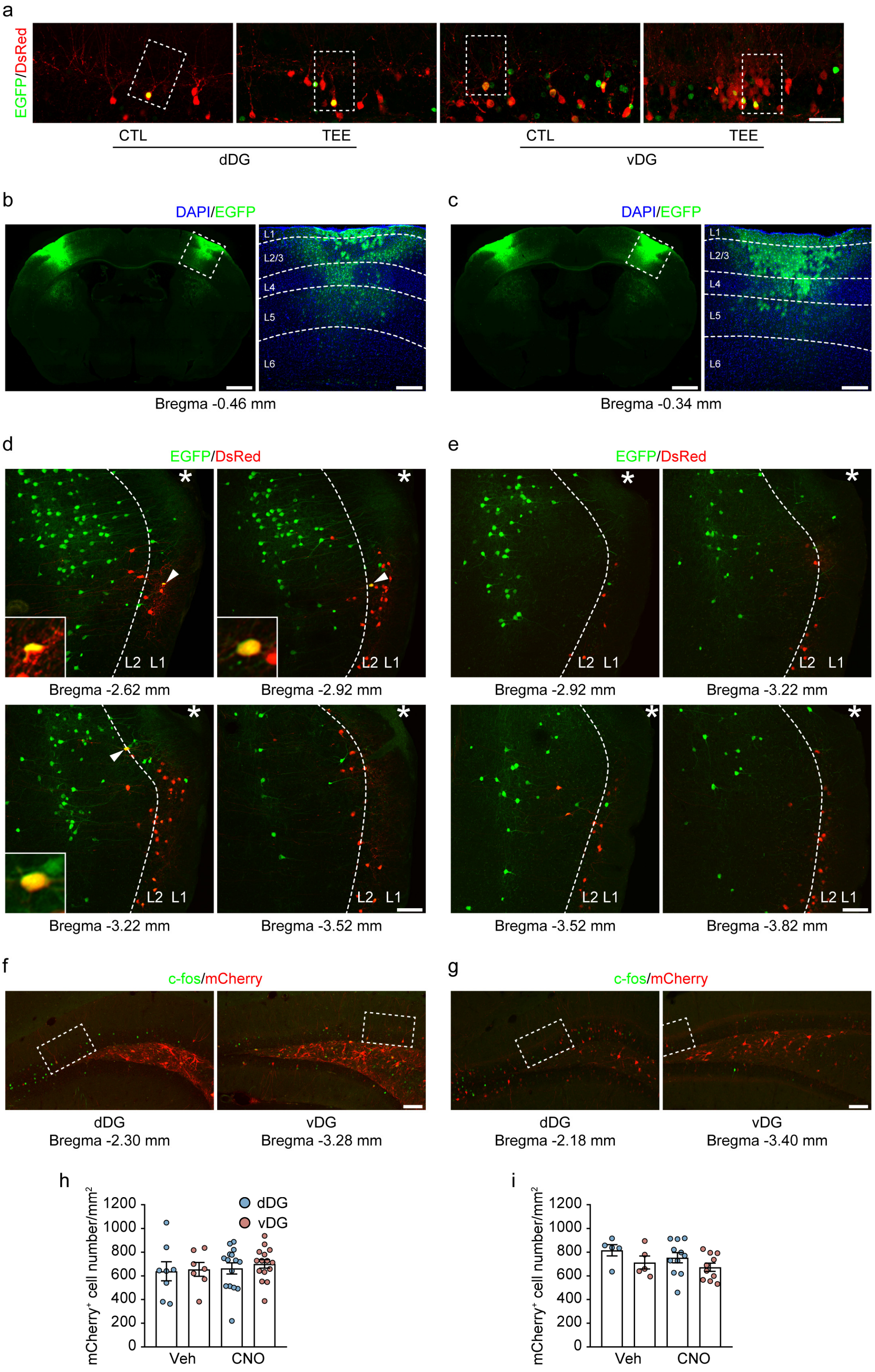

Supplementary Fig. 9 Labeling, tracing and manipulation of tactile experience enrichment-activated DG neurons. **a** Representative images show EGFP and DsRed

double-labeled starter cells in dDG and vDG. Boxed regions are magnified in insets of Fig. 4j. **b**, **c** Representative images show the spreading of the anterograde AAV-Syn-Cre (green) in the S1 of Ai47 mice, which was used to examine S1-innervated lateral entorhinal cortex (LEC) neurons that project to TEE-tagged dDG (**b**) or vDG (**c**) neurons respectively. Boxed regions were imaged at a higher magnification and are presented in right panels. **d**, **e** Representative serial coronal sections show anterogradely labeled S1-innervated cells ($EGFP^+$) and retrogradely labeled (**d**) dDG- or (**e**) vDG-projecting cells ($DsRed^+$) in the LEC. Arrowheads indicate LEC layer 2 cells (magnified in insets) that both received S1 inputs and projected to TEE-tagged dDG neurons. Asterisks indicate the rhinal fissure. **f**, **g** Representative images show c-fos staining and mCherry-labeled dDG or vDG neurons that expressed hM3Dq (**f**) or hM4Di (**g**) in clozapine-N-oxide (CNO)-treated groups. Boxed regions were imaged at a higher magnification and are presented in **Fig. 6c,g** respectively. **h, i** The density of mCherry-labeled TEE-tagged DG neurons in the chemogenetic activation (**h**) and inhibition (**i**) experiments was comparable among vehicle (Veh)- and CNO-treated groups. Scale bars: 50 μm for (**a**), 1 mm for left panels and 200 μm for right panels for (**b** and **c**), and 100 μm for (**d**-**g**). Data are presented as mean ± SEM. Detailed statistics are provided in Supplementary Table 2. Source data are provided as a Source Data file.

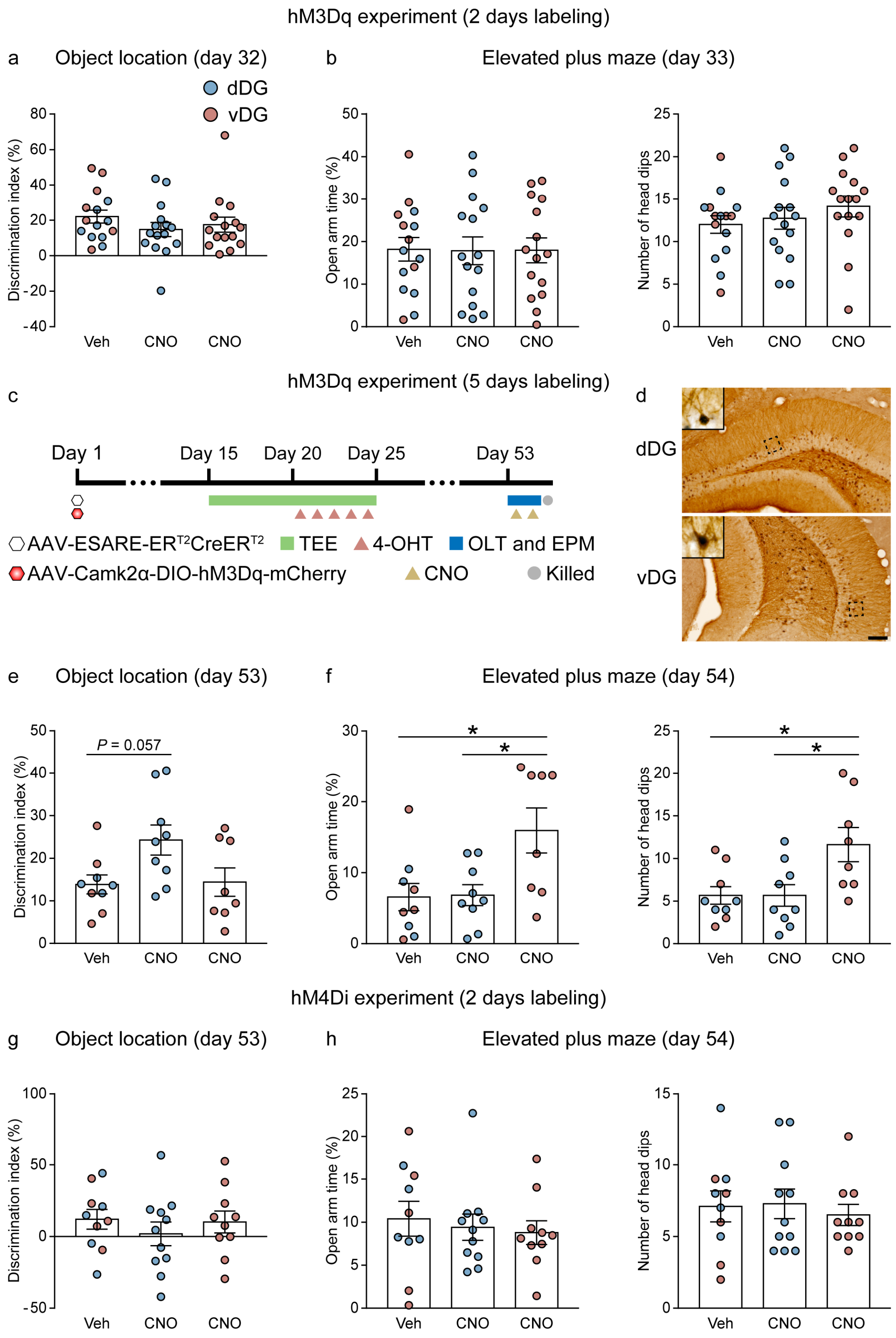

Supplementary Fig. 10 Effects of chemogenetic manipulation of tactile enrichment-tagged dDG or vDG neurons on memory and anxiety. **a**, **b** At 1 week after tactile enrichment,

chemogenetic activation of TEE-tagged dDG or vDG neurons did not affect spatial memory performance (**a**) nor anxiety-related behavior (**b**). **c** The timeline of the validation experiment with a 5-day labeling protocol. OLT, object location task; EPM, elevated plus maze. **d** Representative photomicrographs from the dDG-CNO group (upper panel) and the vDG-CNO group (lower panel) show c-fos (dark blue) and mCherry (brown) immunostaining in dDG and vDG. Boxed regions were imaged at a higher magnification and are presented in insets, showing activated granule cells that were labeled during tactile enrichment. Scale bar = 100 μm. **e, f** At 4 weeks after tactile enrichment, chemogenetic activation of TEE-tagged dDG but not vDG neurons had a trend to improve memory performance (**e**). **A**ctivation of TEE-tagged vDG but not dDG neurons reduced anxiety-related behavior (**f**), as shown by increased percentage of time in open arms and number of head dips in the elevated plus maze compared to control and dDG-CNO mice. **g**, **h** At 4 weeks after tactile enrichment, inactivation of TEE-tagged dDG or vDG neurons did not alter spatial memory performance (**g**) nor anxiety level (**h**). n = 15 mice per group for (**a** and **b**), 8-9 mice per group for (**e** and **f**), and 10-11 mice per group for (**g** and **h**). *$P < 0.05$. Data are presented as mean ± SEM. Detailed statistics are provided in Supplementary Table 2. Source data are provided as a Source Data file.

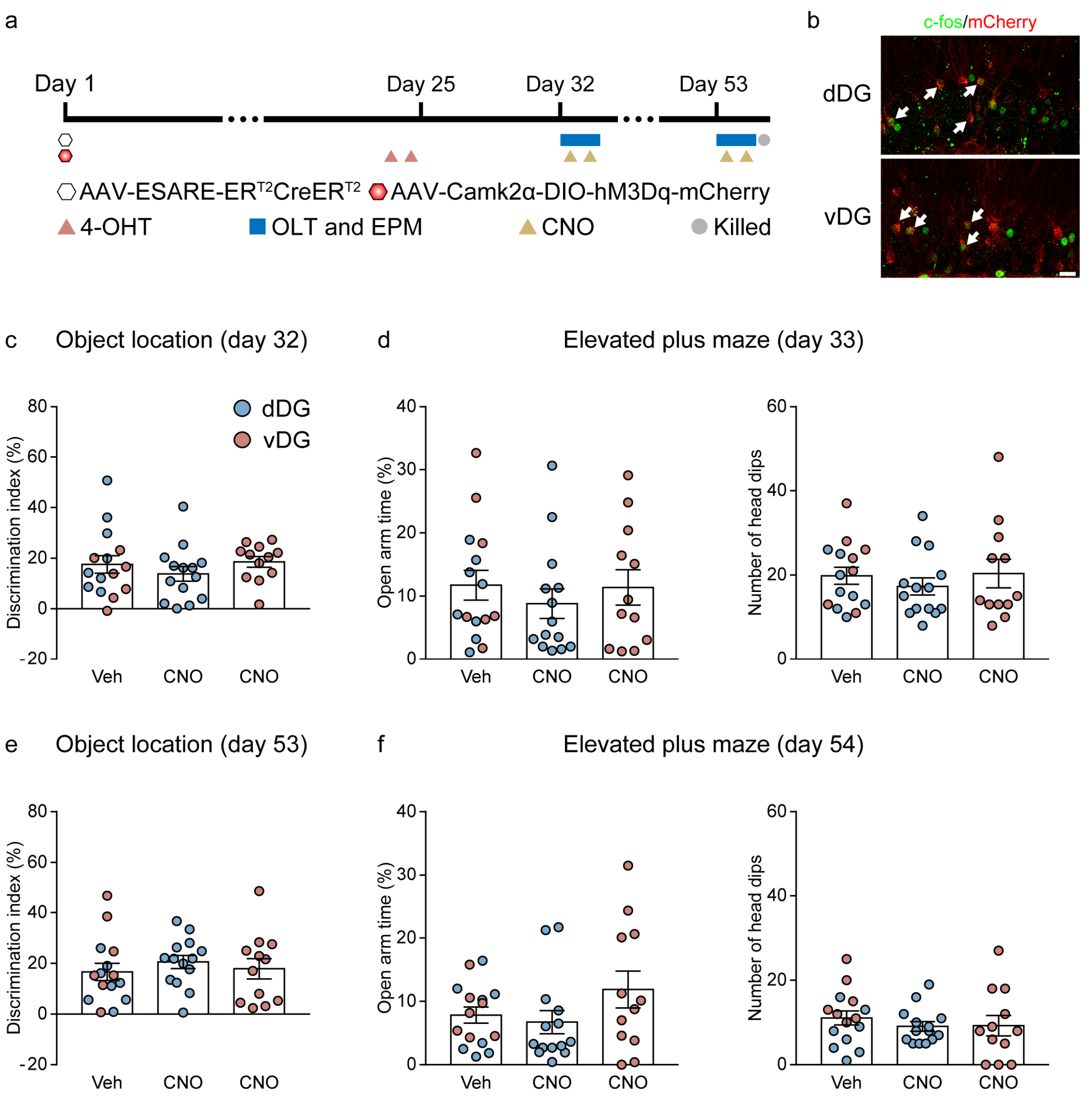

Supplementary Fig. 11 Chemogenetic activation of control DG neurons did not affect memory nor anxiety. **a** The timeline of experiment. The design of experiment was similar to that shown in **Fig. 6b**, except that mice remained singly housed under standard conditions until killed. **b** Representative images from the dDG-CNO group (upper panel) and the vDG-CNO group (lower panel) show c-fos and mCherry immunostaining in dDG and vDG. Arrows indicate chemogenetically activated granule cells that were labeled under home-cage conditions. Scale bar = 20 μm. **c, d** At 8 days after the second 4-OHT injection, chemogenetic activation of control dDG or vDG neurons had no effect on spatial memory performance (**c**)

nor anxiety-related behavior (**d**). **e, f** At 4 weeks after the second 4-OHT injection, chemogenetic activation of control dDG or vDG neurons did not alter memory performance (**e**) nor anxiety level (**f**). n = 12-15 mice per group. Data are presented as mean ± SEM. Detailed statistics are provided in Supplementary Table 2. Source data are provided as a Source Data file.

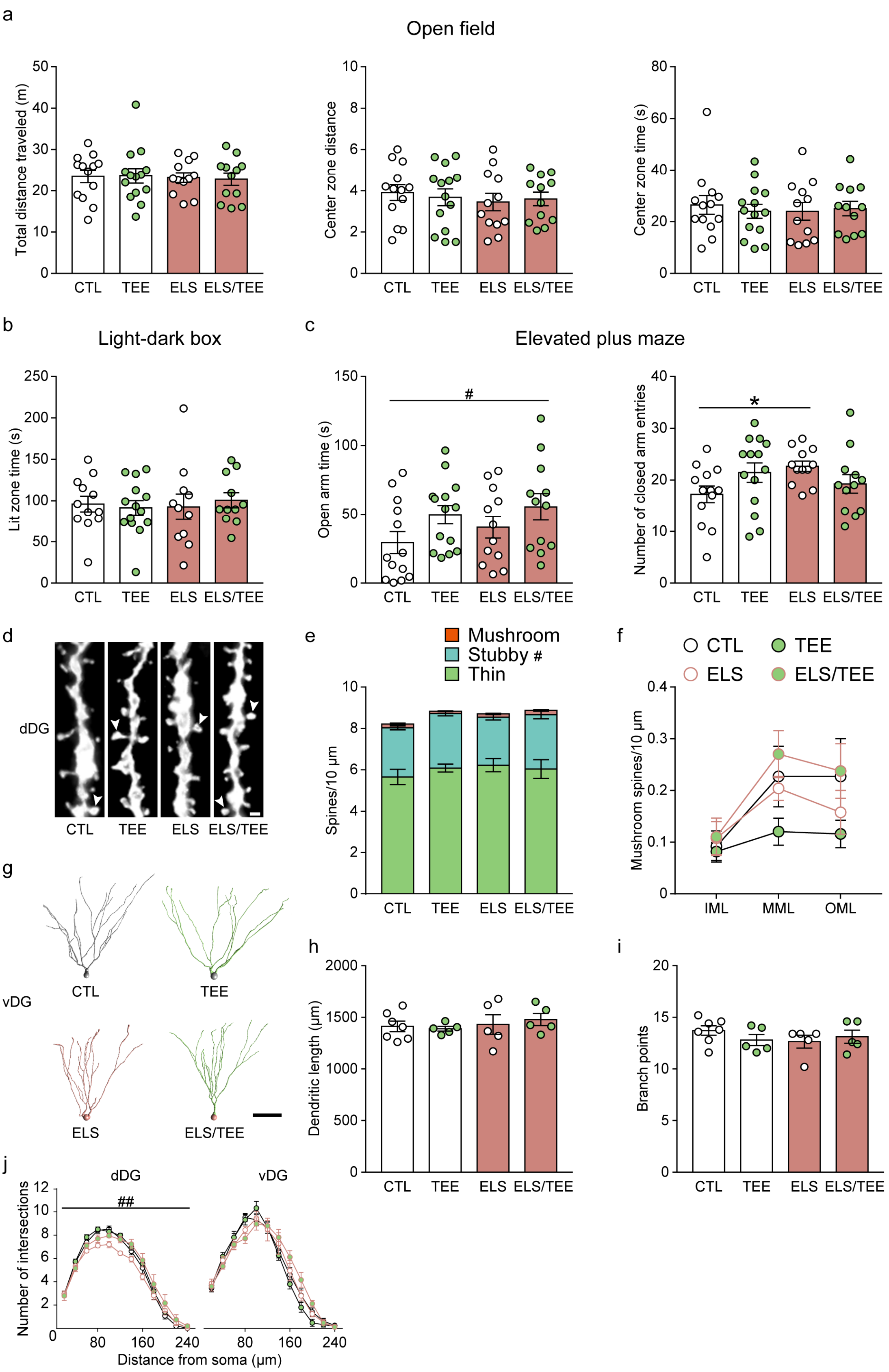
a
Open field
Total distance traveled (m)
Center zone distance
Center zone time (s)
CTL TEE ELS ELS/TEE
b
Light-dark box
Lit zone time (s)
c
Elevated plus maze
Open arm time (s)
Number of closed arm entries
#
*
d
dDG
CTL TEE ELS ELS/TEE
e
Mushroom
Stubby #
Thin
Spines/10 µm
f
CTL
TEE
ELS
ELS/TEE
Mushroom spines/10 µm
IML MML OML
g
vDG
CTL
TEE
ELS
ELS/TEE
h
Dendritic length (µm)
i
Branch points
j
dDG
vDG
##
Number of intersections
Distance from soma (µm)

Supplementary Fig. 12 Effects of early-life stress and adulthood tactile enrichment on anxiety-related behavior and DG neuron structural plasticity. **a** In the open field test, total distance traveled as well as distance traveled and time in center zone were similar among groups. **b** In the light-dark box test, all groups of mice spent comparable time in the brightly lit compartment. **c** In the elevated plus maze test, tactile enrichment increased the time spent in open arms in control and stressed mice. Early-life stressed mice had more visits to closed arms than control mice, whereas the two enrichment groups showed similar performance. **d** Representative images of dendritic segments in the outer molecular layer of the dDG. Arrowheads indicate mushroom spines. Scale bar = 1 μm. **e** Tactile enrichment increased stubby spine density in dDG neurons. Total, mushroom and thin spine density was not affected by early-life stress nor tactile enrichment. **f** Mushroom spine density in the inner, medial and outer molecular layers (abbreviated as IML, MML and OML) of the dDG was comparable among groups. **g** Representative reconstructions of vDG granule cells. Scale bar = 50 μm. **h**, **i** Total dendritic length (**h**) and the number of branch points (**i**) were comparable among groups. **j** Sholl analysis of dendrites of dDG and vDG granule cells. Tactile enrichment increased dendritic complexity of dDG neurons in control and stress mice. No difference in dendritic complexity of vDG neurons was observed among groups. $^{*}P < 0.05$. $^{\#}P < 0.05$, $^{\#\#}P < 0.01$, enrichment effect. Data are presented as mean ± SEM. Detailed statistics are provided in Supplementary Table 2. Source data are provided as a Source Data file.

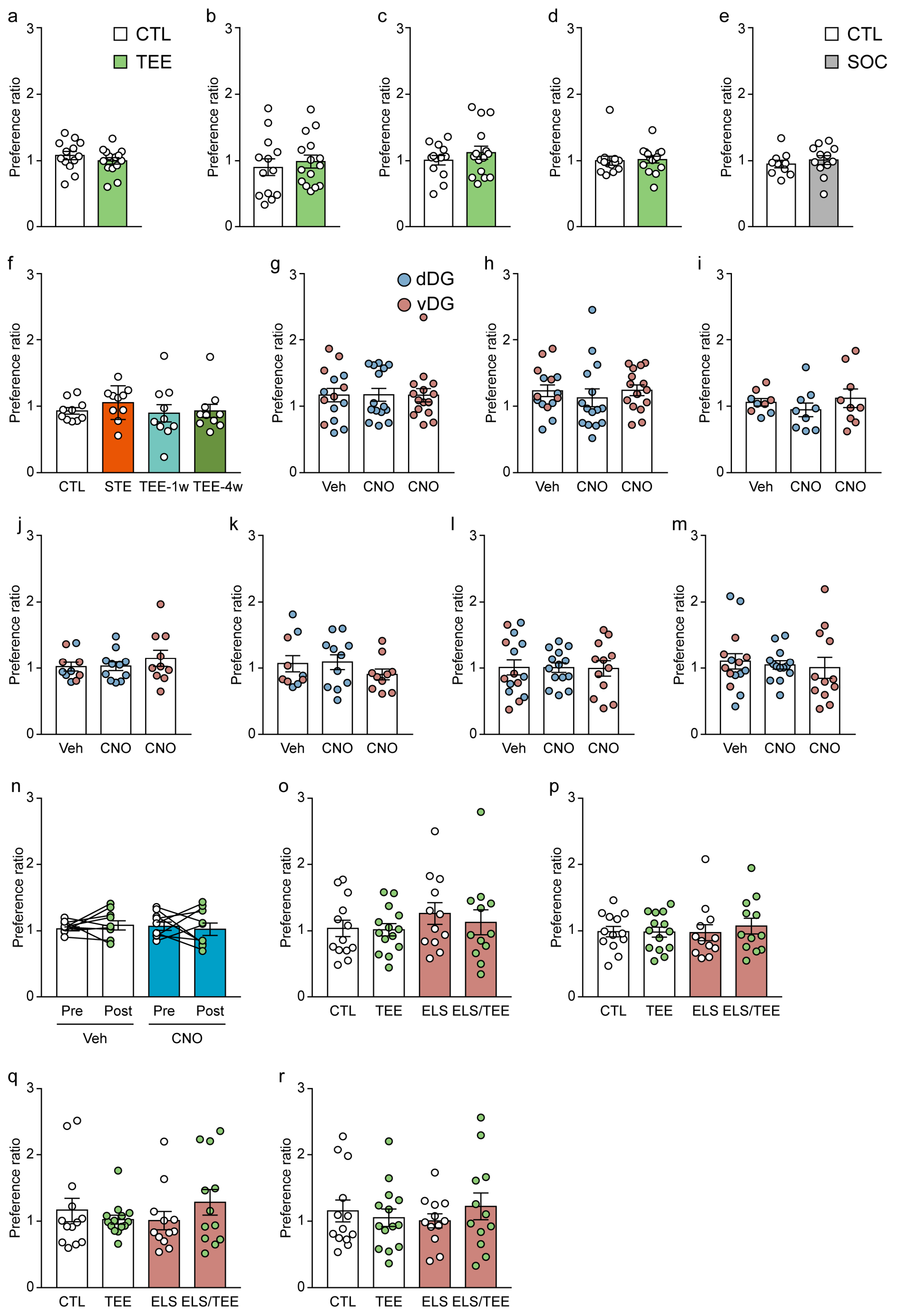
a
b
c
d
e
f
g
h
i
j
k
l
m
n
o
p
q
r
CTL
TEE
CTL
SOC
dDG
vDG
Preference ratio
STE
TEE-1w
TEE-4w
Veh
CNO
Pre
Post
ELS
ELS/TEE

Supplementary Fig. 13 Summary of object preference ratio in the acquisition phases of object recognition tasks in each experiment. In the acquisition phase of the novel object recognition and object location tasks, object preference ratio was calculated as the time with the copy of the familiar or stationary object in the retrieval phase divided by the time with the other object. In the temporal order task, object preference ratio was calculated as the total time with the two objects presented in the first acquisition phase divided by the total time with the two objects presented in the second. In the object-in-place task, object preference ratio was calculated as the total time exploring the copies of two stationary objects in the retrieval phase divided by the total time with the other two objects. **a** Tactile enrichment and object preference in the acquisition phase of the novel object recognition task, corresponding to **Fig. 1g**. **b** Tactile enrichment and object preference in the acquisition phase of the object location task, corresponding to **Fig. 1h**. **c** Tactile enrichment and object preference in the acquisition phases of the temporal order task, corresponding to **Supplementary Fig. 3a**. **d** Tactile enrichment and object preference in the acquisition phase of the object-in-place task, corresponding to **Supplementary Fig. 3b**. **e** Individual versus social housing and object preference in the object location task, corresponding to **Supplementary Fig. 3g**. **f** Varied duration of tactile enrichment on object preference in the object location task, corresponding to **Fig. 3b**. **g** Chemogenetic activation of TEE-tagged DG neurons at 1 week after TEE and object preference in the object location task, corresponding to **Supplementary Fig. 10a**. **h, i** Chemogenetic activation of TEE-tagged DG neurons at 4 weeks after TEE and object preference in the object location task, corresponding to **Fig. 6d** and **Supplementary Fig. 10e**, respectively. **j, k** Chemogenetic inhibition of TEE-tagged DG neurons at 1 week (**j**) or 4

weeks (**k**) after TEE and object preference in the object location task, corresponding to **Fig. 6h** and **Supplementary Fig. 10g**, respectively. **l, m** Chemogenetic activation of control DG neurons at 8 days (**l**) or 4 weeks (**m**) after the last 4-OHT injection and object preference in the object location task, corresponding to **Supplementary Fig. 11c,e**, respectively. **n** Chemogenetic inhibition of S1-innervated LEC neurons and object preference in the object location task, corresponding to **Fig. 7h**. **o-r** Early-life stress, adult tactile enrichment and object preference in the acquisition phase(s) of the novel object recognition (**o**), object location (**p**), temporal order (**q**), and object-in-place tasks (**r**), corresponding to **Fig. 8b-e**, respectively. In any of these experiments, no difference in object preference ratio in the acquisition phase(s) was found between or among groups. Data are presented as mean ± SEM. Detailed statistics are provided in Supplementary Table 2. Source data are provided as a Source Data file.

Supplementary Table 1. A list of viral vectors used in this study.

Virus	Abbreviation	Titer	Source
RV-Ubi-EGFP	Not applicable	1×10^9 viral genomes/ml	Dr. Yan Gu, Zhejiang University School of Medicine
AAV9-ESARE-$ER^{T2}CreER^{T2}$-PEST	AAV-ESARE-$ER^{T2}CreER^{T2}$	1×10^{14} viral genomes/ml	Vigene Biosciences
RABV-EnvA-ΔG-DsRed	RABV-EnvA-ΔG-DsRed	2×10^8 infectious units/ml	BrainVTA Biotechnology
AAV2/9-Ef1α-DIO-His-EGFP-2A-TVA-WPRE-pA	AAV-Ef1α-DIO-EGFP-TVA ("helper")	2×10^{12} viral genomes/ml	BrainVTA Biotechnology
AAV2/9-Ef1α-DIO-RVG-WPRE-pA	AAV-Ef1α-DIO-RVG ("helper")	2×10^{12} viral genomes/ml	BrainVTA Biotechnology
AAV2/1-hSyn-Cre-WPRE-pA	AAV-Syn-Cre	1×10^{13} viral genomes/ml	BrainVTA Biotechnology
AAV9-Camk2α-DIO-hM3Dq-2A-mCherry	AAV-Camk2α-DIO-hM3Dq-mCherry (also hM3Dq)	1×10^{13} viral genomes/ml	Vigene Biosciences
AAV9-Camk2α-DIO-hM4Di-2A-mCherry	AAV-Camk2α-DIO-hM4Di-mCherry (also hM4Di)	1×10^{13} viral genomes/ml	Vigene Biosciences

Supplementary Table 2. Statistical methods, results and sample size related to each figure.

Figure	Statistical method	Factors	Statistical results	*Post hoc* method	*Post hoc* results	Sample size
1c	Two-tailed unpaired t test	Condition	dDG : t_{12} = 5.098, P = 0.0003;<br>vDG : t_{12} = 2.49, P = 0.028;			7 mice per group
1d	Two-tailed unpaired t test	Condition	S1 L2/3: t_{12} = 1.006, P = 0.334;<br>S1 L4: t_{12} = 4.149, P = 0.001;<br>S1 L5: t_{12} = 0.931, P = 0.37;<br>S1 L6: t_{12} = 1.896, P = 0.082;<br>Pir L2: t_{12} = 3.547, P = 0.004;<br>Pir L3: t_{12} = 0.841, P = 0.417;<br>V1 L2/3: t_{12} = 2.958, P = 0.012;<br>V1 L4: $t_{7.413}$ = 1.825, P = 0.108;<br>V1 L5: t_{12} = 2.991, P = 0.011;<br>V1 L6: $t_{7.168}$ = 1.895, P = 0.099;<br>Au1 L2/3: $t_{8.976}$ = 2.016, P = 0.075;<br>Au1 L4: t_{12} = 4.788, P = 0.0004;<br>Au1 L5: t_{12} = 2.501, P = 0.028;<br>Au1 L6: $t_{8.353}$ = 3.159, P = 0.013			
1e	One-way ANOVA	Region	$F_{3,24}$ = 7.554, P = 0.001	Tukey's test	S1 L4 versus V1 L4: P < 0.001;<br>S1 L4 versus Au1 L4: P < 0.05	
1g	Two-tailed unpaired t test	Condition	t_{26} = 2.343, P = 0.027			13 CTL and 15 TEE mice
1h	Two-tailed Mann-Whitney U test	Condition	U = 54.0, P = 0.045			
1i	Two-tailed Mann-Whitney	Condition	U = 53.5, P = 0.043			

	U test					
1j	Two-tailed unpaired *t* test	Condition	$t_{26} = 2.583$, $P = 0.016$			13 CTL and 15 TEE mice
2c	One-way ANOVA	Condition	dDG: $F_{3,21} = 14.33$, $P < 0.0001$<br>vDG: $F_{3,21} = 6.821$, $P = 0.0022$	Tukey's test	**dDG**<br>CTL versus MME: $P < 0.001$;<br>TEE versus MME: $P < 0.001$;<br>SOC versus MME: $P < 0.05$<br>**vDG**<br>CTL versus TEE: $P < 0.05$;<br>CTL versus MME: $P < 0.01$;	6 CTL, 6 TEE, 6 SOC, and 7 MME mice
2d	Kruskal-Wallis test	Condition	dDG: $H = 932.6$, n = 15106, $P < 0.0001$<br>vDG: $H = 232.2$, n = 7636, $P < 0.0001$	Dunn's test	**dDG**<br>CTL versus TEE: $P < 0.001$;<br>CTL versus SOC: $P < 0.001$;<br>CTL versus MME: $P < 0.001$;<br>TEE versus SOC: $P < 0.001$;<br>TEE versus MME: $P < 0.001$;<br>SOC versus MME: $P < 0.001$;<br>**vDG**<br>CTL versus TEE: $P < 0.001$;<br>CTL versus SOC: $P <$	

					0.001;<br>CTL versus MME: *P* < 0.001;<br>TEE versus MME: *P* < 0.001;<br>SOC versus MME: *P* < 0.001;	
3b	One-way ANOVA	Condition	$F_{3,36} = 11.201$, $P = 0.00002$	Tukey's test	CTL versus TEE-1w: *P* < 0.001;<br>TEE-1w versus STE: *P* < 0.01;<br>TEE-1w versus TEE-4w: *P* < 0.001	10 mice per group
3c	One-way ANOVA	Condition	Open arm time%: $F_{3,36} = 4.131$, $P = 0.013$;<br>Head dip number: $F_{3,36} = 7.527$, $P = 0.00049$	Tukey's test	**Open arm time%**<br>CTL versus TEE-1w: *P* < 0.01<br>**Head dip number**<br>CTL versus TEE-1w: *P* < 0.01;<br>TEE-1w versus STE: *P* < 0.05;<br>TEE-1w versus TEE-4w: *P* < 0.01	
3f	One-way ANOVA	Condition	dDG: $F_{2,18} = 1.039$, $P = 0.374$;<br>vDG: $F_{2,18} = 3.623$, $P = 0.0476$	Tukey's test	**vDG**<br>CTL versus TEE: *P* < 0.05	7 mice per group
3g	Kruskal-Wallis test	Condition	dDG: $H = 40.29$, n = 4017, $P < 0.0001$<br>vDG: $H = 40.0$, n = 1690, $P < 0.0001$	Dunn's test	**dDG**<br>CTL versus TEE: *P* < 0.05;<br>CTL versus TEE-1w: *P* < 0.001;<br>TEE versus TEE-1w: *P*	

					< 0.001;<br>**vDG**<br>CTL versus TEE: *P* < 0.001;<br>TEE versus TEE-1w: *P* < 0.001;	
4c	Two-tailed unpaired *t* test	Condition	dDG: $t_{14} = 0.037$, $P = 0.971$;<br>vDG: $t_{14} = 0.597$, $P = 0.56$			8 mice per group
4d	Two-tailed unpaired *t* test	Condition	dDG: $t_{14} = 2.205$, $P = 0.045$;<br>vDG: $t_{14} = 1.36$, $P = 0.195$			
4f	Two-tailed unpaired *t* test	Condition	dDG: $t_{14} = 1.718$, $P = 0.108$;<br>vDG: $t_{14} = 2.168$, $P = 0.048$			
4g	One-way repeated measures ANOVA	Condition × sublayer	dDG: $F_{1,14} = 20.123$, $P = 0.0005$;<br>vDG: $F_{1,14} = 17.074$, $P = 0.001$	Two-tailed unpaired *t* test	**dDG**<br>IML: $t_{14} = 2.157$, $P = 0.049$;<br>MML: $t_{14} = 3.314$, $P = 0.005$;<br>OML: $t_{14} = 4.535$, $P = 0.0005$<br>**vDG**<br>IML: $t_{8.115} = 1.425$, $P = 0.192$;<br>MML: $t_{10.131} = 2.441$, $P = 0.034$;<br>OML: $t_{14} = 2.91$, $P = 0.011$	
4k	Two-tailed unpaired *t* test	Condition	dDG: $t_9 = 0.306$, $P = 0.766$;<br>vDG: $t_7 = 1.277$, $P = 0.242$			6 CTL-dDG, 5 TEE-dDG, 4 CTL-vDG, and 5 TEE-vDG mice
4l	Two-tailed unpaired *t* test	Condition	dDG: $t_9 = 2.433$, $P = 0.0378$;<br>vDG: $t_7 = 2.525$, $P = 0.0395$			
6d	One-way	Condition	$F_{2,42} = 7.482$, $P = 0.002$	Tukey's test	dDG-CNO versus Veh:	15 mice per

	ANOVA				$P < 0.01$;<br>dDG-CNO versus vDG-CNO: $P < 0.01$	group
6e	One-way ANOVA	Condition	Open arm time%: $F_{2,42} = 22.237$, $P = 0.0000003$;<br>Head dip number: $F_{2,42} = 10.547$, $P = 0.0002$	Tukey's test	**Open arm time%**<br>vDG-CNO versus Veh: $P < 0.001$;<br>vDG-CNO versus dDG -CNO: $P < 0.01$<br>**Head dip number**<br>vDG-CNO versus Veh: $P < 0.001$;<br>vDG-CNO versus dDG -CNO: $P < 0.001$	15 mice per group
6h	Discrimination index: one-way ANOVA; Time with object%: two-tailed paired *t* test	Discriminat -ion index: condition; Time with object%: object	**Discrimination index**<br>$F_{2,28} = 0.384$, $P = 0.685$<br>**Time with object%**<br>Veh: 58.42 ± 2.29 with relocated versus 41.58 ± 2.29 with familiar, $t_9 = 3.682$, $P = 0.005$;<br>dDG-CNO: 55.38 ± 3.7 with relocated versus 44.62 ± 3.7 with familiar, $t_{10} = 1.453$, $P = 0.177$;<br>vDG-CNO: 58.65 ± 2.61 with relocated versus 41.35 ± 2.61 with familiar, $t_9 = 3.318$, $P = 0.009$			10 Veh, 11 dDG-CNO and 10 vDG-CNO mice
6i	One-way ANOVA	Condition	Open arm time%: $F_{2,28} = 3.976$, $P = 0.0302$;<br>Head dip number: $F_{2,28} = 9.593$, $P = 0.0007$	Tukey's test	**Open arm time%**<br>vDG-CNO versus Veh: $P = 0.073$;<br>vDG-CNO versus dDG -CNO: $P < 0.05$<br>**Head dip number**<br>vDG-CNO versus Veh: $P < 0.01$;	

					vDG-CNO versus dDG -CNO: $P < 0.01$	
7c	One-way ANOVA	Condition	S1 L2/3: $F_{2,69} = 45.1$, $P < 0.0001$;<br>S1 L4: $F_{2,69} = 159.7$, $P < 0.0001$;<br>S1 L5: $F_{2,69} = 38.77$, $P < 0.0001$;<br>S1 L6: $F_{2,69} = 24.66$, $P < 0.0001$;<br>LEC L2: $F_{2,69} = 8.993$, $P = 0.0003$;<br>LEC L3: $F_{2,69} = 3.466$, $P = 0.0368$;<br>LEC L5: $F_{2,69} = 3.827$, $P = 0.0265$;<br>LEC L6: $F_{2,69} = 5.257$, $P = 0.0075$;<br>dDG: $F_{2,69} = 7.651$, $P = 0.001$;<br>vDG: $F_{2,69} = 6.392$, $P = 0.0045$;	Tukey's test	**S1 L2/3**<br>CTL versus Chronic: $P < 0.001$;<br>Acute versus Chronic: $P < 0.001$;<br>**S1 L4**<br>CTL versus Acute: $P < 0.001$;<br>CTL versus Chronic: $P < 0.001$;<br>Acute versus Chronic: $P < 0.001$;<br>**S1 L5**<br>CTL versus Acute: $P < 0.001$;<br>CTL versus Chronic: $P < 0.001$;<br>**S1 L6**<br>CTL versus Acute: $P = 0.0061$;<br>CTL versus Chronic: $P < 0.001$;<br>Acute versus Chronic: $P < 0.001$;<br>**LEC 2**<br>CTL versus Acute: $P < 0.01$;<br>CTL versus Chronic: $P < 0.001$;<br>**LEC 3**	8 images for S1, LEC and dDG, and 4 images for vDG per mouse; 3 mice per group

					CTL versus Acute: P = 0.0458;<br>**LEC 5**<br>Acute versus Chronic: P = 0.0218;<br>**LEC 6**<br>Acute versus Chronic: P = 0.0052;<br>**dDG**<br>CTL versus Acute: P < 0.001;<br>Acute versus Chronic: P = 0.0205;<br>**vDG**<br>CTL versus Acute: P < 0.01;<br>CTL versus Chronic: P = 0.0124;	
7e	Kruskal-Wallis test	Condition	dDG: H = 24.02, n = 2590, P < 0.0001<br>vDG: H = 38.7, n = 1652, P < 0.0001	Dunn's test	**dDG**<br>CTL versus Acute: P < 0.001;<br>CTL versus Chronic: P = 0.0071;<br>Acute versus Chronic: P = 0.0349;<br>**vDG**<br>CTL versus Acute: P < 0.001;<br>CTL versus Chronic: P < 0.001;	3 mice per group
7h	Two-way ANOVA	Enrichment × treatment	Enrichment: $F_{1,32}$ = 0.1691, P = 0.6836;<br>Treatment: $F_{1,32}$ = 2.377, P = 0.1329;	Tukey's test	Post-Veh versus Post-CNO: P = 0.0352	9 mice per group

			Interaction: $F_{1,32} = 6.283$, $P = 0.0175$			
7i	Two-way ANOVA	Enrichment × treatment	Enrichment: $F_{1,32} = 0.06231$, $P = 0.8045$; Treatment: $F_{1,32} = 2.999$, $P = 0.0929$; Interaction: $F_{1,32} = 4.819$, $P = 0.0355$	Tukey's test	Post-Veh versus Post-CNO: $P = 0.0429$	9 mice per group
8b	Two-way ANOVA	Stress × enrichment	Stress: $F_{1,47} = 15.012$, $P = 0.00033$; Enrichment: $F_{1,47} = 34.546$, $P = 0.0000004$; Interaction: $F_{1,47} = 7.856$, $P = 0.0073$	Tukey's test	All CTL versus ELS: $P < 0.001$; All ELS/TEE versus ELS: $P < 0.001$	13 CTL, 14 TEE, 12 ELS, and 12 ELS/TEE mice
8c	Two-way ANOVA	Stress × enrichment	Stress: $F_{1,47} = 18.459$, $P = 0.000087$; Enrichment: $F_{1,47} = 43.359$, $P = 0.00000003$; Interaction: $F_{1,47} = 15.341$, $P = 0.00029$	Tukey's test		
8d	Two-way ANOVA	Stress × enrichment	Stress: $F_{1,47} = 9.349$, $P = 0.0037$; Enrichment: $F_{1,47} = 9.774$, $P = 0.003$; Interaction: $F_{1,47} = 21.677$, $P = 0.00003$	Tukey's test		
8e	Two-way ANOVA	Stress × enrichment	Stress: $F_{1,47} = 9.58$, $P = 0.0033$; Enrichment: $F_{1,47} = 9.197$, $P = 0.0039$; Interaction: $F_{1,47} = 12.4$, $P = 0.00097$	Tukey's test		
8f	Two-way ANOVA	Stress × enrichment	Stress: $F_{1,44} = 1.875$, $P = 0.178$; Enrichment: $F_{1,44} = 2.362$, $P = 0.132$; Interaction: $F_{1,44} = 4.135$, $P = 0.048$ (statistical outliers were excluded)	Tukey's test	CTL versus ELS: $P < 0.05$; ELS/TEE versus ELS: $P < 0.05$	
8g	Two-way ANOVA	Stress × enrichment	Stress: $F_{1,47} = 0.929$, $P = 0.34$; Enrichment: $F_{1,47} = 4.797$, $P = 0.034$; Interaction: $F_{1,47} = 0.131$, $P = 0.719$			
8i	Two-way ANOVA	Stress × enrichment	Stress: $F_{1,18} = 2.732$, $P = 0.116$; Enrichment: $F_{1,18} = 7.476$, $P = 0.014$; Interaction: $F_{1,18} = 2.127$, $P = 0.162$			7 CTL, 5 TEE, 5 ELS, and 5 ELS/TEE mice
8j	Two-way ANOVA	Stress × enrichment	Stress: $F_{1,18} = 9.569$, $P = 0.006$; Enrichment: $F_{1,18} = 2.837$, $P = 0.109$; Interaction: $F_{1,18} = 7.757$, $P = 0.012$	Tukey's test	CTL versus ELS: $P < 0.001$; ELS/TEE versus ELS:	

					$P < 0.05$	
8l	Two-way ANOVA	Stress × enrichment	**Total**<br>Stress: $F_{1,18} = 0.008$, $P = 0.931$;<br>Enrichment: $F_{1,18} = 5.588$, $P = 0.0295$;<br>Interaction: $F_{1,18} = 0.238$, $P = 0.632$<br>**Mushroom**<br>Stress: $F_{1,18} = 1.704$, $P = 0.208$;<br>Enrichment: $F_{1,18} = 6.707$, $P = 0.019$;<br>Interaction: $F_{1,18} = 1.333$, $P = 0.264$<br>**Stubby**<br>Stress: $F_{1,18} = 1.924$, $P = 0.182$;<br>Enrichment: $F_{1,18} = 1.296$, $P = 0.2699$;<br>Interaction: $F_{1,18} = 0.031$, $P = 0.863$<br>**Thin**<br>Stress: $F_{1,18} = 0.084$, $P = 0.775$;<br>Enrichment: $F_{1,18} = 4.85$, $P = 0.041$;<br>Interaction: $F_{1,18} = 0.209$, $P = 0.653$			7 CTL, 5 TEE, 5 ELS, and 5 ELS/TEE mice
8m	Two-way repeated measures ANOVA	Stress × enrichment × sublayer	Stress: $F_{1,18} = 1.396$, $P = 0.253$;<br>Enrichment: $F_{1,18} = 8.428$, $P = 0.009$;<br>Interaction: $F_{1,18} = 0.88$, $P = 0.361$			
S2c	Two-way ANOVA	Condition × zone	Condition: $F_{3,42} = 1.773$, $P = 0.167$;<br>Zone: $F_{1,42} = 53.008$, $P = 5.7839E-9$;<br>Interaction: $F_{3,42} = 0.322$, $P = 0.809$			7 CTL, 6 Nestlet, 6 Beads, and 6 Beads/Nestlet mice
S2d	Two-way ANOVA	Condition × zone	Condition: $F_{3,42} = 0.506$, $P = 0.68$;<br>Zone: $F_{1,42} = 26.116$, $P = 0.000007$;<br>Interaction: $F_{3,42} = 1.026$, $P = 0.391$			
S2e	Two-way ANOVA	Condition × time	Condition: $F_{3,42} = 0.845$, $P = 0.477$;<br>Time: $F_{1,42} = 5.116$, $P = 0.029$;<br>Interaction: $F_{3,42} = 1.348$, $P = 0.272$			
S2f	Two-way ANOVA	Condition × time	Condition: $F_{1,20} = 10.74$, $P = 0.004$;<br>Time: $F_{1,20} = 5.441$, $P = 0.03$;			6 mice per group

			Interaction: $F_{1,20} = 0.527$, $P = 0.476$			
S2g	Pearson correlation analysis	S1 L4 c-fos^{+} neuron density and distance in curtain zone	CTL: $r = 0.281$, $P = 0.589$; Nestlet: $r = 0.374$, $P = 0.535$; Beads: $r = -0.515$, $P = 0.374$; Beads/Nestlet: $r = 0.836$, $P = 0.038$			6 mice per group
S2i	Two-way ANOVA	Condition × time	Condition: $F_{1,20} = 6.56$, $P = 0.019$; Time: $F_{1,20} = 0.575$, $P = 0.457$; Interaction: $F_{1,20} = 0.024$, $P = 0.878$			6 mice
S2j	Two-way ANOVA	Condition × time	Condition: $F_{1,20} = 2.788$, $P = 0.111$; Time: $F_{1,20} = 0.383$, $P = 0.543$; Interaction: $F_{1,20} = 0.024$, $P = 0.879$			
S3a	Two-tailed unpaired *t* test	Condition	$t_{26} = 0.764$, $P = 0.452$			13 CTL and 15 TEE mice
S3b	Two-tailed unpaired *t* test	Condition	$t_{26} = 1.571$, $P = 0.128$			
S3c	Two-tailed unpaired *t* test	Condition	$t_{26} = 2.723$, $P = 0.011$			
S3d	Two-tailed unpaired *t* test	Condition	$t_{26} = 0.995$, $P = 0.329$			
S3f	One-way ANOVA	Condition	dDG: $F_{3,21} = 1.107$, $P = 0.3683$; vDG: $F_{3,21} = 5.53$, $P = 0.0059$	Tukey's test	**vDG** CTL versus One row: $P = 0.0428$; Half row versus One row: $P = 0.0341$; Half row versus Three rows: $P = 0.0397$	6 CTL, 6 Half row, 6 One row, and 7 Three rows
S3g	Two-tailed unpaired *t* test	Condition	$t_{20} = 0.7123$, $P = 0.4845$			10 CTL and 12 SOC mice
S3h	Two-tailed unpaired *t* test	Condition	$t_{20} = 0.2395$, $P = 0.8132$; $t_{20} = 0.1953$, $P = 0.8471$			

S4e	One-way ANOVA	Condition	BF L2/3: $F_{3,21} = 5.407$, $P = 0.0065$;<br>BF L4: $F_{3,21} = 4.799$, $P = 0.0106$;<br>BF L5: $F_{3,21} = 5.333$, $P = 0.0069$;<br>BF L6: $F_{3,21} = 6.828$, $P = 0.0022$	Tukey's test	**BF L2/3**<br>CTL versus TEE, $P < 0.05$;<br>CTL versus SOC, $P < 0.05$<br>CTL versus MME, $P < 0.01$<br>**BF L4**<br>CTL versus TEE, $P < 0.05$;<br>CTL versus SOC, $P < 0.05$<br>CTL versus MME, $P < 0.05$<br>**BF L5**<br>CTL versus TEE, P < 0.05;<br>CTL versus MME, P < 0.01<br>**BF L6**<br>CTL versus TEE, P < 0.05;<br>CTL versus MME, P < 0.01	6 CTL, 6 TEE, 6 SOC, and 7 MME mice
S4f	One-way ANOVA	Condition	FL L2/3: $F_{3,21} = 1.791$, $P = 0.1798$;<br>FL L4: $F_{3,21} = 1.022$, $P = 0.4029$;<br>FL L5: $F_{3,21} = 3.481$, $P = 0.034$;<br>FL L6: $F_{3,21} = 4.247$, $P = 0.0171$	Tukey's test	**FL L5**<br>CTL versus MME, $P < 0.05$<br>**FL L6**<br>CTL versus MME, $P < 0.05$	
S4g	One-way ANOVA	Condition	HL L2/3: $F_{3,21} = 1.307$, $P = 0.2985$;<br>HL L4: $F_{3,21} = 0.8117$, $P = 0.5016$;	Tukey's test	**HL L5**<br>CTL versus MME, $P <$	

			HL L5: $F_{3,21} = 3.13$, $P = 0.0474$;<br>HL L6: $F_{3,21} = 3.301$, $P = 0.0403$		0.05<br>**HL L6**<br>CTL versus MME, $P <$ 0.05	
S4h	One-way ANOVA	Condition	Sh L2/3: $F_{3,21} = 1.503$, $P = 0.2429$;<br>Sh L4: $F_{3,21} = 0.9179$, $P = 0.4493$;<br>Sh L5: $F_{3,21} = 3.5$, $P = 0.0335$;<br>Sh L6: $F_{3,21} = 3.185$, $P = 0.0449$	Tukey's test	**Sh L5**<br>CTL versus MME, $P <$ 0.05<br>**Sh L6**<br>CTL versus MME, $P <$ 0.05	6 CTL, 6 TEE, 6 SOC, and 7 MME mice
S4i	One-way ANOVA	Condition	Tr L2/3: $F_{3,21} = 4.389$, $P = 0.0151$;<br>Tr L4: $F_{3,21} = 3.175$, $P = 0.0454$;<br>Tr L5: $F_{3,21} = 4.736$, $P = 0.0112$;<br>Tr L6: $F_{3,21} = 4.035$, $P = 0.0206$	Tukey's test	**Tr L2/3**<br>CTL versus TEE, $P <$ 0.05;<br>CTL versus MME, $P <$ 0.05<br>**Tr L4**<br>CTL versus MME, $P <$ 0.05<br>**Tr L5**<br>CTL versus TEE, $P <$ 0.05;<br>CTL versus MME, $P <$ 0.05<br>**Tr L6**<br>CTL versus TEE, $P <$ 0.05;<br>CTL versus MME, $P <$ 0.05	
S5a	Two-tailed unpaired t test	Condition	dDG: $t_{10} = 1.58$, $P = 0.145$;<br>vDG: $t_{10} = 1.358$, $P = 0.204$			6 mice per group
S5b	Two-tailed unpaired t test	Condition	dDG: $t_{10} = 2.011$, $P = 0.072$;<br>vDG: $t_{10} = 0.235$, $P = 0.819$			

S5c	Two-tailed unpaired *t* test	Condition	dDG: $t_{10} = 0.003$, $P = 0.998$; vDG: $t_{10} = 0.251$, $P = 0.807$			6 mice per group
S5d	Two-tailed unpaired *t* test	Condition	dDG: $t_{10} = 0.328$, $P = 0.75$; vDG: $t_{10} = 0.876$, $P = 0.402$			
S5e	Two-tailed unpaired *t* test	Condition	dDG: $t_{10} = 0.591$, $P = 0.568$; vDG: $t_{10} = 0.648$, $P = 0.532$			
S5f	Two-tailed unpaired *t* test	Condition	dDG: $t_{10} = 0.801$, $P = 0.442$; vDG: $t_{10} = 0.75$, $P = 0.471$			
S6c	One-way repeated measures ANOVA	Condition × distance	dDG: $F_{1,73} = 0.004$, $P = 0.951$; vDG: $F_{1,34} = 0.448$, $P = 0.508$			38 CTL dDG, 37 TEE dDG, 22 CTL vDG, and 14 TEE vDG neurons from 7 CTL and 8 TEE mice
S6d	Two-tailed unpaired *t* test	Condition	dDG: $t_{73} = 0.002$, $P = 0.999$; vDG: $t_{34} = 0.168$, $P = 0.868$			
S6e	Two-tailed unpaired *t* test	Condition	dDG: $t_{73} = 1.915$, $P = 0.059$; vDG: $t_{34} = 1.095$, $P = 0.281$			
S6g	Two-tailed unpaired *t* test	Condition	dDG: $t_{68} = 0.812$, $P = 0.42$; vDG: $t_{57} = 0.501$, $P = 0.618$			41 CTL dDG, 29 TEE dDG, 29 CTL vDG, and 30 TEE vDG dendrites; 6 mice per group
S6h	One-way repeated measures ANOVA	Condition × sublayer	dDG: $F_{1,68} = 0.00044$, $P = 0.983$; vDG: $F_{1,57} = 1.674$, $P = 0.201$			
S6i	Two-tailed unpaired *t* test	Condition	dDG: $t_{10} = 0.6461$, $P = 0.5328$; vDG: $t_{10} = 0.7328$, $P = 0.4805$			6 mice per group
S6j	Two-tailed unpaired *t* test	Condition	dDG: $t_{10} = 0.6666$, $P = 0.5201$; vDG: $t_{10} = 0.5034$, $P = 0.6256$			
S7a	One-way repeated measures ANOVA	Condition × distance	dDG: $F_{1,14} = 0.014$, $P = 0.907$; vDG: $F_{1,14} = 0.197$, $P = 0.664$			8 mice per group

S7d	One-way ANOVA	Condition	dDG: $F_{3,21} = 0.7839$, $P = 0.5163$;<br>vDG: $F_{3,21} = 0.08873$, $P = 0.9654$			6 CTL, 6 TEE, 6 SOC, and 7 MME mice
S7e	One-way repeated measures ANOVA	Condition	dDG: $F_{3,21} = 1.565$, $P = 0.228$;<br>vDG: $F_{3,21} = 0.265$, $P = 0.85$			
S7f	One-way ANOVA	Condition	dDG: $F_{3,21} = 1.368$, $P = 0.2798$;<br>vDG: $F_{3,21} = 1.104$, $P = 0.3695$			
S7h	One-way ANOVA	Condition	**dDG**<br>Total: $F_{3,21} = 7.202$, $P = 0.0017$;<br>Mushroom: $F_{3,21} = 4.363$, $P = 0.015$;<br>Stubby: $F_{3,21} = 2.386$, $P = 0.098$;<br>Thin: $F_{3,21} = 4.396$, $P = 0.015$<br><br>**vDG**<br>Total: $F_{3,21} = 7.748$, $P = 0.0011$;<br>Mushroom: $F_{3,21} = 11.353$, $P = 0.00012$;<br>Stubby: $F_{3,21} = 1.117$, $P = 0.365$;<br>Thin: $F_{3,21} = 6.325$, $P = 0.\ 003$	Tukey's or Tamhane's test	**dDG Total**<br>CTL versus MME, $P < 0.01$;<br>SOC versus MME, $P < 0.05$<br>**dDG Mushroom**<br>CTL versus TEE, $P < 0.05$<br>**dDG Thin**<br>CTL versus MME, $P < 0.05$;<br>TEE versus MME, $P < 0.05$<br>SOC versus MME, $P < 0.05$<br><br>**vDG Total**<br>CTL versus MME, $P < 0.05$;<br>TEE versus MME, $P < 0.01$<br>SOC versus MME, $P < 0.01$<br>**vDG Mushroom**	

					CTL versus TEE, *P* < 0.01<br>TEE versus SOC, *P* < 0.001<br>**vDG Thin**<br>TEE versus MME, *P* < 0.01<br>SOC versus MME, *P* < 0.01	
S7i	Overall difference: one-way repeated measures ANOVA; sublayer difference: One-way ANOVA	Overall difference: condition × sublayer; sublayer difference: condition	Overall difference in dDG: $F_{3,21} = 4.142$, $P = 0.019$;<br>Overall difference in vDG: $F_{3,21} = 11.218$, $P = 0.00013$<br><br>dDG IML: $F_{3,21} = 2.757$, $P = 0.068$;<br>dDG MML: $F_{3,21} = 3.099$, $P = 0.049$;<br>dDG OML: $F_{3,21} = 2.198$, $P = 0.118$<br><br>vDG IML: $F_{3,21} = 15.602$, $P = 0.000$;<br>vDG MML: $F_{3,21} = 6.761$, $P = 0.002$;<br>vDG OML: $F_{3,21} = 6.259$, $P = 0.003$	Tukey's or Tamhane's test	**dDG MML**<br>CTL versus TEE, *P* < 0.05<br><br>**vDG IML**<br>CTL versus TEE, *P* < 0.001<br>CTL versus MME, *P* < 0.01<br>TEE versus SOC, *P* < 0.001<br>SOC versus MME, *P* < 0.01<br>**vDG MML**<br>CTL versus TEE, *P* < 0.01<br>TEE versus SOC, *P* < 0.01<br>**vDG OML**<br>CTL versus TEE, *P* < 0.01<br>TEE versus SOC, *P* < 0.01	6 CTL, 6 TEE, 6 SOC, and 7 MME mice

S8d	Two-tailed unpaired *t* test	Condition	S1 L2/3: $t_4 = 3.104$, $P = 0.036$;<br>S1 L4: $t_4 = 5.276$, $P = 0.006$;<br>S1 L5: $t_4 = 3.403$, $P = 0.074$;<br>S1 L6: $t_4 = 2.0$, $P = 0.184$			3 mice
S8g	One-way ANOVA	Condition	$F_{3,23} = 1.242$, $P = 0.3173$			6 CTL, 7 TED1, 7 TED5, and 7 TED10 mice
S8h	One-way ANOVA	Condition	$F_{3,23} = 3.41$, $P = 0.0345$	Tukey's test	CTL versus TED5, $P < 0.05$	
S9h	Two-way ANOVA	Condition × treatment	Condition: $F_{1,41} = 0.2408$, $P = 0.6263$;<br>Treatment: $F_{1,41} = 0.4072$, $P = 0.5269$;<br>Interaction: $F_{1,41} = 0.03952$, $P = 0.8434$			8 dDG-Veh, 7 vDG-Veh, 15 dDG-CNO, and 15 vDG-CNO mice
S9i	Two-way ANOVA	Condition × treatment	Condition: $F_{1,27} = 3.655$, $P = 0.0666$;<br>Treatment: $F_{1,27} = 1.14$, $P = 0.2951$;<br>Interaction: $F_{1,27} = 0.05508$, $P = 0.8162$			5 dDG-Veh, 5 vDG-Veh, 11 dDG-CNO, and 10 vDG-CNO mice
S10a	One-way ANOVA	Condition	$F_{2,42} = 0.864$, $P = 0.429$			15 mice per group
S10b	One-way ANOVA	Condition	Open arm time%: $F_{2,42} = 0.003$, $P = 0.997$;<br>Head dip number: $F_{2,42} = 0.825$, $P = 0.445$			
S10e	One-way ANOVA	Condition	$F_{2,23} = 3.71$, $P = 0.0401$	Tukey's test	dDG-CNO versus Veh: $P = 0.057$;<br>dDG-CNO versus vDG-CNO: $P = 0.085$	9 Veh, 9 dDG-CNO and 8 vDG-CNO mice
S10f	One-way ANOVA	Condition	Open arm time%: $F_{2,23} = 5.55$, $P = 0.011$;<br>Head dip number: $F_{2,23} = 5.463$, $P = 0.011$	Tukey's test	**Open arm time%**<br>vDG-CNO versus Veh: $P < 0.05$;<br>vDG-CNO versus dDG	

					-CNO: $P < 0.05$<br>**Head dip number**<br>vDG-CNO versus Veh: $P < 0.05$;<br>vDG-CNO versus dDG -CNO: $P < 0.05$	
S10g	One-way ANOVA	Condition	$F_{2,28} = 0.514$, $P = 0.604$			10 Veh, 11 dDG-CNO and 10 vDG-CNO mice
S10h	One-way ANOVA	Condition	Open arm time%: $F_{2,28} = 0.233$, $P = 0.794$;<br>Head dip number: $F_{2,28} = 0.175$, $P = 0.841$			
S11c	One-way ANOVA	Condition	$F_{2,38} = 0.6926$, $P = 0.5065$			15 Veh, 14 dDG-CNO and 12 vDG-CNO mice
S11d	One-way ANOVA	Condition	Open arm time%: $F_{2,38} = 0.4247$, $P = 0.657$;<br>Head dip number: $F_{2,38} = 0.4368$, $P = 0.6493$			
S11e	One-way ANOVA	Condition	$F_{2,38} = 0.3827$, $P = 0.6846$			
S11f	One-way ANOVA	Condition	Open arm time%: $F_{2,38} = 1.73$, $P = 0.1909$;<br>Head dip number: $F_{2,38} = 0.4215$, $P = 0.6591$			
S12a	Two-way ANOVA	Stress × enrichment	**Total distance**<br>Stress: $F_{1,47} = 0.128$, $P = 0.722$;<br>Enrichment: $F_{1,47} = 0.007$, $P = 0.933$;<br>Interaction: $F_{1,47} = 0.032$, $P = 0.859$<br>**Center distance**<br>Stress: $F_{1,47} = 0.477$, $P = 0.493$;<br>Enrichment: $F_{1,47} = 0.01$, $P = 0.921$;<br>Interaction: $F_{1,47} = 0.238$, $P = 0.628$			13 CTL, 14 TEE, 12 ELS, and 12 ELS/TEE mice

			**Center time**<br>Stress: $F_{1,47} = 0.052$, $P = 0.821$;<br>Enrichment: $F_{1,47} = 0.042$, $P = 0.838$;<br>Interaction: $F_{1,47} = 0.311$, $P = 0.5798$			
S12b	Two-way ANOVA	Stress × enrichment	Stress: $F_{1,44} = 0.075$, $P = 0.786$;<br>Enrichment: $F_{1,44} = 0.023$, $P = 0.879$;<br>Interaction: $F_{1,44} = 0.334$, $P = 0.567$<br>(statistical outliers were excluded)			13 CTL, 14 TEE, 12 ELS, and 12 ELS/TEE mice
S12c	Two-way ANOVA	Stress × enrichment	**Open arm time**<br>Stress: $F_{1,47} = 1.113$, $P = 0.297$;<br>Enrichment: $F_{1,47} = 4.821$, $P = 0.033$;<br>Interaction: $F_{1,47} = 0.113$, $P = 0.739$<br>**Closed arm entries**<br>Stress: $F_{1,47} = 0.993$, $P = 0.324$;<br>Enrichment: $F_{1,47} = 0.057$, $P = 0.812$;<br>Interaction: $F_{1,47} = 5.425$, $P = 0.024$	Tukey's test	**Closed arm entries**<br>CTL versus ELS: $P < 0.05$	
S12e	Two-way ANOVA	Stress × enrichment	**Total**<br>Stress: $F_{1,18} = 0.482$, $P = 0.497$;<br>Enrichment: $F_{1,18} = 1.107$, $P = 0.307$;<br>Interaction: $F_{1,18} = 0.325$, $P = 0.575$<br>**Mushroom**<br>Stress: $F_{1,18} = 0.974$, $P = 0.337$;<br>Enrichment: $F_{1,18} = 0.128$, $P = 0.725$;<br>Interaction: $F_{1,18} = 2.38$, $P = 0.14$<br>**Stubby**<br>Stress: $F_{1,18} = 0.073$, $P = 0.79$;<br>Enrichment: $F_{1,18} = 4.703$, $P = 0.044$;<br>Interaction: $F_{1,18} = 0.024$, $P = 0.878$<br>**Thin**<br>Stress: $F_{1,18} = 0.533$, $P = 0.475$;<br>Enrichment: $F_{1,18} = 0.112$, $P = 0.742$;<br>Interaction: $F_{1,18} = 0.733$, $P = 0.403$			7 CTL, 5 TEE, 5 ELS, and 5 ELS/TEE mice

S12f	Two-way repeated measures ANOVA	Stress × enrichment × sublayer	Stress: $F_{1,18} = 0.818$, $P = 0.378$;<br>Enrichment: $F_{1,18} = 0.102$, $P = 0.753$;<br>Interaction: $F_{1,18} = 2.311$, $P = 0.146$			7 CTL, 5 TEE, 5 ELS, and 5 ELS/TEE mice
S12h	Two-way ANOVA	Stress × enrichment	Stress: $F_{1,18} = 0.761$, $P = 0.394$;<br>Enrichment: $F_{1,18} = 0.04$, $P = 0.844$;<br>Interaction: $F_{1,18} = 0.33$, $P = 0.573$			
S12i	Two-way ANOVA	Stress × enrichment	Stress: $F_{1,18} = 0.453$, $P = 0.51$;<br>Enrichment: $F_{1,18} = 0.15$, $P = 0.703$;<br>Interaction: $F_{1,18} = 1.546$, $P = 0.23$			
S12j	Two-way repeated measures ANOVA	Stress × enrichment × distance	**dDG**<br>Stress: $F_{1,18} = 3.79$, $P = 0.067$;<br>Enrichment: $F_{1,18} = 8.319$, $P = 0.00988$;<br>Interaction: $F_{1,18} = 2.467$, $P = 0.134$<br>**vDG**<br>Stress: $F_{1,18} = 0.25$, $P = 0.623$;<br>Enrichment: $F_{1,18} = 0.004$, $P = 0.952$;<br>Interaction: $F_{1,18} = 0.487$, $P = 0.494$			
S13a	Two-tailed unpaired *t* test	Condition	$t_{26} = 1.036$, $P = 0.31$			13 CTL and 15 TEE
S13b	Two-tailed unpaired *t* test	Condition	$t_{26} = 0.552$, $P = 0.586$			
S13c	Two-tailed unpaired *t* test	Condition	$t_{26} = 0.9033$, $P = 0.3746$			
S13d	Two-tailed unpaired *t* test	Condition	$t_{26} = 0.1993$, $P = 0.8436$			
S13e	Two-tailed unpaired *t* test	Condition	$t_{20} = 0.673$, $P = 0.508$			10 CTL and 12 SOC
S13f	One-way ANOVA	Condition	$F_{3,36} = 0.542$, $P = 0.657$			10 mice per group
S13g	One-way ANOVA	Condition	$F_{2,42} = 0.001$, $P = 0.999$			15 mice per group

S13h	One-way ANOVA	Condition	$F_{2,42} = 0.374$, $P = 0.69$			15 mice per group
S13i	One-way ANOVA	Condition	$F_{2,24} = 0.704$, $P = 0.504$			9 mice per group
S13j	One-way ANOVA	Condition	$F_{2,28} = 0.639$, $P = 0.535$			10 Veh, 11 dDG-CNO and 10 vDG-CNO mice
S13k	One-way ANOVA	Condition	$F_{2,28} = 0.867$, $P = 0.431$			
S13l	One-way ANOVA	Condition	$F_{2,38} = 0.005$, $P = 0.995$			15 Veh, 14 dDG-CNO and 12 vDG-CNO mice
S13m	One-way ANOVA	Condition	$F_{2,38} = 0.178$, $P = 0.838$			
S13n	Two-way ANOVA	Enrichment × treatment	Enrichment: $F_{1,32} = 0.01805$, $P = 0.894$; Treatment: $F_{1,32} = 0.8017$, $P = 0.3773$; Interaction: $F_{1,32} = 0.01805$, $P = 0.894$			9 mice per group
S13o	Two-way ANOVA	Stress × enrichment	Stress: $F_{1,47} = 1.411$, $P = 0.241$; Enrichment: $F_{1,47} = 0.29$, $P = 0.593$; Interaction: $F_{1,47} = 0.156$, $P = 0.695$			13 CTL, 14 TEE, 12 ELS, and 12 ELS/TEE mice
S13p	Two-way ANOVA	Stress × enrichment	Stress: $F_{1,47} = 0.152$, $P = 0.698$; Enrichment: $F_{1,47} = 0.218$, $P = 0.643$; Interaction: $F_{1,47} = 0.293$, $P = 0.591$			
S13q	Two-way ANOVA	Stress × enrichment	Stress: $F_{1,47} = 0.116$, $P = 0.735$; Enrichment: $F_{1,47} = 0.196$, $P = 0.66$; Interaction: $F_{1,47} = 2.017$, $P = 0.162$			
S13r	Two-way ANOVA	Stress × enrichment	Stress: $F_{1,47} = 0.005$, $P = 0.945$; Enrichment: $F_{1,47} = 0.14$, $P = 0.71$; Interaction: $F_{1,47} = 1.078$, $P = 0.304$			
