# Supplementary material for: Tactile modulation of memory and anxiety requires dentate granule cells along the dorsoventral axis
Source: Nat Commun. 2020 Nov 27;11:6045. doi: 10.1038/s41467-020-19874-8 (PMC7695841; doi:10.1038/s41467-020-19874-8)
Supplement: Supplementary file 3 — Description of Additional Supplementary Files [file 41467_2020_19874_MOESM3_ESM.pdf]

Description of Additional Supplementary Files

Supplementary Movie 1: Multimodal enrichment cage setup. Mice are group-housed in a large cage containing a nest box, a running wheel, plastic tubes, Lego bricks, and Nestlets.

Supplementary Movie 2: Tactile enrichment cage setup. A singly housed mouse is exploring a standard-sized cage provided with a bead curtain and Nestlets.
