# Supplementary material for: Tactile modulation of memory and anxiety requires dentate granule cells along the dorsoventral axis
Source: Nat Commun. 2020 Nov 27;11:6045. doi: 10.1038/s41467-020-19874-8 (PMC7695841; doi:10.1038/s41467-020-19874-8)
Supplement: Supplementary file 6 — Reporting Summary [file 41467_2020_19874_MOESM6_ESM.pdf]

nature research

Corresponding author(s): Xiao-Dong Wang
Last updated by author(s): Oct 26, 2020

Reporting Summary

Nature Research wishes to improve the reproducibility of the work that we publish. This form provides structure for consistency and transparency in reporting. For further information on Nature Research policies, see Authors & Referees and the Editorial Policy Checklist .

Statistics

For all statistical analyses, confirm that the following items are present in the figure legend, table legend, main text, or Methods section.

n/a	Confirmed	
☐	☒	The exact sample size (*n*) for each experimental group/condition, given as a discrete number and unit of measurement
☐	☒	A statement on whether measurements were taken from distinct samples or whether the same sample was measured repeatedly
☐	☒	The statistical test(s) used AND whether they are one- or two-sided<br>*Only common tests should be described solely by name; describe more complex techniques in the Methods section.*
☐	☒	A description of all covariates tested
☐	☒	A description of any assumptions or corrections, such as tests of normality and adjustment for multiple comparisons
☐	☒	A full description of the statistical parameters including central tendency (e.g. means) or other basic estimates (e.g. regression coefficient) AND variation (e.g. standard deviation) or associated estimates of uncertainty (e.g. confidence intervals)
☐	☒	For null hypothesis testing, the test statistic (e.g. *F*, *t*, *r*) with confidence intervals, effect sizes, degrees of freedom and *P* value noted<br>*Give P values as exact values whenever suitable.*
☒	☐	For Bayesian analysis, information on the choice of priors and Markov chain Monte Carlo settings
☒	☐	For hierarchical and complex designs, identification of the appropriate level for tests and full reporting of outcomes
☐	☒	Estimates of effect sizes (e.g. Cohen's *d*, Pearson's *r*), indicating how they were calculated

Our web collection on statistics for biologists contains articles on many of the points above.

Software and code

Policy information about availability of computer code

Data collection

We have listed all softwares used for data collection in the Methods section.
Behavioral data were collected using the automated video-tracking system ANY-maze 5.14 (Stoelting). Immunofluorescence images were obtained with an Olympus IX83–FV3000 laser scanning confocal microscope. For spatial pattern analysis of c-fos+ neurons in the DG, density analysis of c-fos+ neurons in S1 subregions and trans-synaptic tracing experiments, images were obtained using an Olympus VS120 virtual slide scanning system. Other images were captured with an Olympus BX61 microscope and the cellSens Dimension 2.2 software.
For spatial pattern analysis of c-fos+ neurons in the DG and detailed quantification of c-fos+ neurons in S1 subregions, images were segmented by the U-Net Segmentation plugin of ImageJ (https://sites.imagej.net/Falk/) with a model fine-tuned from a pertained cell segmentation model (https://lmb.informatik.uni-freiburg.de/lmbsoft/unet/). The fine-tuned model for c-fos+ cell segmentation is deposited on GitHub (https://github.com/unetzjuser/Finetuned-unet-model-for-neuron-detection). Fluorescent images of dendritic spines were deconvolved using the AutoQuant X3.0.4 software (Media Cybernetics, registration number: 3532).

Data analysis

We have listed all softwares used for data analysis in the Methods section.
Excel 2016 (Microsoft) was used to organize data. Behavioral data were analyzed using ANY-maze 5.14 (Stoelting). Dendrites and spines of dentate granule cells were analyzed using the NeuronStudio software (Rodriguez, A., Ehlenberger, D.B., Hof, P.R. & Wearne, S.L. Rayburst sampling, an algorithm for automated three-dimensional shape analysis from laser scanning microscopy images. Nat. Protoc. 1, 2152-2161, 2006). Other image data were analyzed by ImageJ 1.52e with custom settings. The R codes for spatial pattern analysis and examples are deposited on GitHub (https://github.com/unetzjuser/Spatial-pattern-analysis-of-neurons). SPSS 22.0 (SPSS), GraphPad Prism 8.0 (GraphPad Software) and R 3.6.3 (http://www.r-project.org/) were used to perform statistical analyses.

For manuscripts utilizing custom algorithms or software that are central to the research but not yet described in published literature, software must be made available to editors/reviewers. We strongly encourage code deposition in a community repository (e.g. GitHub). See the Nature Research guidelines for submitting code & software for further information.

Data

Policy information about availability of data

All manuscripts must include a data availability statement. This statement should provide the following information, where applicable:

- Accession codes, unique identifiers, or web links for publicly available datasets
- A list of figures that have associated raw data
- A description of any restrictions on data availability

The data that support the findings of this study are available from the corresponding author upon reasonable request. Source data are provided with this paper. The fine-tuned model for c-fos+ cell segmentation is deposited on GitHub (https://github.com/unetzjuser/Finetuned-unet-model-for-neuron-detection).

Field-specific reporting

Please select the one below that is the best fit for your research. If you are not sure, read the appropriate sections before making your selection.

☒ Life sciences ☐ Behavioural & social sciences ☐ Ecological, evolutionary & environmental sciences

For a reference copy of the document with all sections, see nature.com/documents/nr-reporting-summary-flat.pdf

Life sciences study design

All studies must disclose on these points even when the disclosure is negative.

Sample size	Sample sizes were chosen based on published work and reported power analyses in this field (e.g., Dedic N et al., 2018, Nat Neurosci, PMID: 29786085; Gehrlach DA et al., 2019, Nat Neurosci, PMID: 31455886; Daviu N et al., 2020, Nat Neurosci, PMID: 32066984), and are similar to or exceeding those in our previous publications (e.g., Li JT et al., 2017, Cell Rep, PMID: 29069596; Liu WZ et al., 2020, Nat Commun, PMID: 32376858). In general, sample sizes are based on the smallest number required to reach statistical significance in order to reduce unnecessary use of animals.
Data exclusions	In the light-dark box test (presented in Fig. 8f and Supplementary Fig. 12b), statistical outliers with values that fell beyond two standard deviations from the mean were excluded from the final analysis. This criterion has been established previously (e.g, Wang et al, 2012). Exclusion of these outliers is stated in Supplementary Table 2. For all other figures, all data were included.
Replication	To establish the tactile experience enrichment model, only one cohort of mice was used because all subsequent experiments were performed based on this model, which yielded stable and reproducible results. For the effects of chemogenetic activation of TEE-tagged DG neurons on memory and anxiety-related behavior, we replicated our findings using an alternative labeling strategy (5 days instead of 2 days of labeling) with a separate cohort of mice (these data are presented in Supplementary Fig. 10c-f). Other behavioral experiments (including chemogenetic manipulation) were repeated at least once with different cohorts of mice by different lab members. For all other (including immunostaining and morphology) data, each experiment was repeated at least once using different sample series from same animals by different lab members. All replication attempts were successful.
Randomization	Adult mice were randomly assigned to different experimental groups. For early-life stress experiments, mice from each litter were randomly assigned to the control or the tactile enrichment group in adulthood.
Blinding	For TEE-tagged DG neuron labeling, tracing and manipulation experiments, images could not be analyzed blindly because virus(es)-infected brain regions were obvious to investigators. For all other experiments and analyses, investigators were blind to experimental conditions.

Reporting for specific materials, systems and methods

We require information from authors about some types of materials, experimental systems and methods used in many studies. Here, indicate whether each material, system or method listed is relevant to your study. If you are not sure if a list item applies to your research, read the appropriate section before selecting a response.

Materials & experimental systems

n/a	Involved in the study	
☐	☒	Antibodies
☒	☐	Eukaryotic cell lines
☒	☐	Palaeontology
☐	☒	Animals and other organisms
☒	☐	Human research participants
☒	☐	Clinical data

Methods

n/a	Involved in the study	
☒	☐	ChIP-seq
☒	☐	Flow cytometry
☒	☐	MRI-based neuroimaging

Antibodies

Antibodies used	The following primary antibodies were used: rabbit anti-c-fos (1:10000, 226003, Synaptic Systems), guinea pig anti-c-fos (1:1000, 226004, Synaptic Systems), rabbit anti-minichromosome maintenance complex component 2 (MCM2; 1:1000, 4007, Cell Signaling), goat anti-doublecortin (1:1000, sc-8066, Santa Cruz), rabbit anti-calbindin (1:2000, CB-38a, SWANT), mouse anti-calbindin (1:2000, 300, SWANT), rabbit anti-vesicular GABA transporter (VGAT; 1:2000, 131013, Synaptic Systems), rabbit anti-mineralocorticoid receptor (MR; 1:500, ab64457, Abcam), rabbit anti-glucocorticoid receptor (GR; 1:1000, sc-1004, Santa Cruz), rabbit anti-early growth response 1 (Egr1; 1:1000, MA5-15008, Invitrogen), rabbit anti-EGFP (1:2000, sc-8334, Santa Cruz), mouse anti-reelin (1:5000, MAB5364, Millipore), and mouse anti-mCherry (1:5000, E022110-01, EarthOx).<br><br>The following secondary antibodies were used: donkey anti-rabbit Alexa Fluor 488 (1:2000, A-21206, Invitrogen), donkey anti-guinea pig Alexa Fluor 488 (1:2000, 706-545-148, Jackson ImmunoResearch), donkey anti-goat Alexa Fluor 488 (1:2000, A-11055, Invitrogen), donkey anti-mouse Alexa Fluor 488 (1:2000, A-21202, Invitrogen), donkey anti-rabbit Alexa Fluor 594 (1:2000, A-21207, Invitrogen), donkey anti-mouse Alexa Fluor 594 (1:2000, A-21203, Invitrogen), peroxidase-conjugated goat anti-rabbit (undiluted, ZB-2301, Zhongshan Golden Bridge), and peroxidase-conjugated goat anti-mouse (undiluted, ZB-2305, Zhongshan Golden Bridge).
Validation	The rabbit anti-c-fos has been validated by the supplier in mouse brain sections (https://www.sysy.com/products/c-fos/facts-226003.php) and by us (e.g., Yu et al. 2018, doi.org/10.1016/j.neuropharm.2018.04.002).<br>The guinea pig anti-c-fos has been validated by the supplier in mouse hippocampal sections (https://www.sysy.com/product/226004#gallery-4) and previous studies (e.g., Song et al. 2019, doi: 10.1126/sciadv.aat3210).<br>The rabbit anti-minichromosome maintenance complex component 2 has been validated by us in mouse hippocampal sections (e.g., Wang et al. 2017, doi:10.1038/tp.2017.196).<br>The goat anti-doublecortin has been validated by previous studies in mouse brain sections (e.g., James et al. 2016, doi: 10.1002/glia).<br>The rabbit anti-calbindin has been validated by many studies and us in mouse hippocampal sections (e.g., Wang et al. 2017, doi:10.1038/tp.2017.196; Li et al. 2017, doi.org/10.1016/j.celrep.2017.10.006).<br>The mouse anti-calbindin has been validated by many studies and us in mouse hippocampal sections (e.g., Li et al. 2017, doi.org/10.1016/j.celrep.2017.10.006).<br>The rabbit anti-vesicular GABA transporter has been validated by the supplier using knockout tissue (https://www.sysy.com/products/vgat/facts-131013.php) and by us in mouse hippocampal sections (e.g., Yu et al. 2018, doi.org/10.1016/j.neuropharm.2018.04.002).<br>The rabbit anti-mineralocorticoid receptor has been validated by our collaborators in rat hippocampal tissue (Li et al. 2017, doi.org/10.3389/fnmol.2017.00025).<br>The rabbit anti-glucocorticoid receptor has been validated by previous studies in rat hippocampal tissue (e.g., Mitic et al. 2017, doi.org/10.1016/j.bbr.2017.07.014).<br>The rabbit anti-early growth response 1 has been validated by the supplier in PC12 cells (https://www.thermofisher.com/cn/zh/antibody/product/EGR1-Antibody-clone-T-160-5-Monoclonal/MA5-15008) and previous studies (e.g., Zhang et al. 2018, doi: 10.1038/s41422-018-0092-9).<br>The rabbit anti-enhanced green fluorescent protein has been validated by us in mouse hippocampal sections (e.g., Wang et al. 2017, doi:10.1038/tp.2017.196).<br>The mouse anti-reelin has been validated by the supplier in mouse brain lysates (https://www.abcam.com/reelin-antibody-g10-ab78540.html) and by previous studies in mouse brain tissue (e.g., Nisimov et al. 2018, doi.org/10.1016/j.neuroscience.2018.05.021).<br>The mouse anti-mCherry has been validated by us in mouse hippocampal sections (e.g., Yu et al. 2018, doi.org/10.1016/j.neuropharm.2018.04.002).<br><br>Secondary antibodies have been validated by the suppliers (https://www.thermofisher.com, https://www.jacksonimmuno.com, http://www.zsbio.com) and us (Wang et al. 2017, doi:10.1038/tp.2017.196; Li et al. 2017, doi.org/10.1016/j.celrep.2017.10.006; Yu et al. 2018, doi.org/10.1016/j.neuropharm.2018.04.002).

Animals and other organisms

Policy information about studies involving animals; ARRIVE guidelines recommended for reporting animal research

Laboratory animals	Adult male and female C57BL/6N mice (SLAC Laboratories), Rosa26-GFP (Ai47) reporter mice (Allen Institute for Brain Science) and Scnn1a-Cre mice (stock number 009613, Jackson Laboratory) aged 2-2.5 months at the start of experiments were used. For early-life stress experiments, male C57BL/6N offspring were stressed on postnatal days 2 to 9 and examined on postnatal days 75-95.
Wild animals	This study did not involve wild animals.
Field-collected samples	This study did not involve samples collected from the field.
Ethics oversight	The experiments were approved by the Animal Advisory Committee at Zhejiang University and performed in compliance with the National Institute of Health's Guide for the Use and Care of Laboratory Animals.

Note that full information on the approval of the study protocol must also be provided in the manuscript.
